# Career sacrifice for an LGBTQ*-friendly work environment? a choice experiment to investigate the job preferences of LGBTQ* people

**DOI:** 10.1371/journal.pone.0296419

**Published:** 2024-06-24

**Authors:** Zaza Zindel, Lisa de Vries

**Affiliations:** Faculty of Sociology, Bielefeld University, Bielefeld, Germany; Spanish Scientific Research Council, SPAIN

## Abstract

Recent research in economics and sociology demonstrates the existence of significant occupational segregation by sexual orientation and gender identity and differences in a range of labor market outcomes, such as hiring chances, earnings, and leadership positions. In this paper, we examine one possible cause of these differences that is associated with the disadvantaged position of sexual and gender minorities in the labor market: LGBTQ* individuals’ choices aimed at avoiding possible discrimination. This paper examines LGBTQ* people’s relative importance of income, time, promotion prospects, an LGBTQ*-friendly work environment, and diversity management in the decision for or against a job. Based on a discrete choice experiment conducted in a large online sample recruited through social media in Germany (N = 4,507), an LGBTQ*-friendly work climate accounted, on average, for 33.8 percent of respondents’ decisions which is comparable with the relative importance of income. Overtime, a diversity management on company level and promotion prospects are less important in the job decision process of LGBTQ* people. While the results show only small differences by sexual orientation, they show group-specific preferences by gender identity. An LGBTQ*-friendly work climate is more important for cisgender women of the LGBTQ* community and gender minorities than for cisgender men of the LGBTQ* community. In contrast, income is less important for gender minorities and cisgender women of the LGBTQ* community than for cisgender men of the LGBTQ* community.

## Introduction

In many Western countries, policies are being implemented to increase the legal equality and social acceptance of LGBTQ* (lesbian, gay, bisexual, transgender, queer, and other non-cisgender and non-heterosexual) people and thus to improve their lives and living conditions [1–3]. While sexual and gender minorities (we use the term sexual and gender minorities in this paper synonymously for LGBTQ* people) are still highly victimized in many countries worldwide [4,5], attitudes toward homosexuality, same-sex relationships, and transgender people have improved in many Western countries since the 1980s [1,2,5], and anti-discrimination laws have been passed to increase their legal protection (e.g., in the workplace) [3]. A growing number of countries have also legalized same-sex marriage and adoption for same-sex couples, and some have introduced a third gender/sex option in official documents [3]. In Germany, the context analyzed in this paper, the legal equality and social acceptance of LGBTQ* people have increased since the adoption of the General Equal Treatment Act (AGG) in 2006, which prohibits discrimination in work life, and since the legalization of same-sex marriage in 2017 [3].

Despite these improvements, LGBTQ* people report discrimination in many areas of life [1,6; for Germany, see 7] and are at high risk of being victims of violence and harassment [4,6]. Experimental research provides strong evidence of discrimination against LGBTQ* people in hiring processes [8–10], and a large body of research documents that LGBTQ* people are more disadvantaged in their labor market outcomes than the rest of the population [11,12; for Germany, see 7,13]. Moreover, recent research provides evidence of occupational segregation by sexual orientation and gender identity: LGBTQ* people work in different occupations and industry sectors than cisgender heterosexual people [7,14–16]. One key assumption researchers have proposed to explain this occupational segregation is that it is driven partly by LGBTQ* people’s choices aimed at avoiding discrimination in particular areas of the labor market. This assumption is underpinned by research showing that LGBTQ* people differ not only in their labor market outcomes but also in their job preferences and aspirations as well as career interests [17–20]. Empirical and theoretical research on the effect of discrimination on job preferences and aspirations has found evidence of such effects in other minority groups (e.g., ethnic minorities) [21,22]. Nevertheless, there are only a few studies on the avoidance of discrimination by sexual and gender minorities, and the link between LGBTQ* minority status and career decisions remains almost unexplored.

This article focuses on the attempts of LGBTQ* people to avoid a discriminatory working environment by sacrificing income and promotion prospects and accepting more unpaid overtime hours for an LGBTQ*-friendly work climate and diversity management in the workplace. Although recent research provides evidence of the high importance of an LGBTQ*-friendly workplace [7], it remains unclear to what extent LGBTQ* people are willing to sacrifice career opportunities and labor market outcomes for a work environment that protects them from discrimination. This indirect effect of discrimination on careers is virtually unexplored in the research to date. Based on a discrete choice experiment conducted in a large sample of LGBTQ* people in Germany recruited through social media (N = 4,507), this article examines the following research question: To what extent are LGBTQ* people willing to sacrifice career opportunities and labor market outcomes for an LGBTQ*-friendly work climate and diversity management? Moreover, we examine differences within the LGBTQ* community by sexual orientation and gender identity.

## Background

### Labor market discrimination against LGBTQ* people

Based on the theoretical concept of heteronormativity, discrimination, and marginalization against LGBTQ* people are grounded in an implicit moral system that assumes a gender binary and heterosexuality in our society. Sexualities (e.g., lesbian, gay, bisexual) and gender identities (e.g., transgender, intersex) that do not fit into this system are considered as ‘deviant’ and disadvantaged in many aspects of society compared to ‘normative’ sexualities and genders (heterosexual cisgender men and women) [see e.g., 23,24]. In line with this, previous research on labor market outcomes of LGBTQ* people offer evidence of discrimination against this group [1,2].

Since the 1990s, income inequality based on sexual orientation has been examined in numerous studies and recent research has found that gay men earn less than heterosexual men, whereas there are mixed results for lesbian women compared to heterosexual women [25]. Other studies have shown that bisexual women and men tend to earn less than heterosexual individuals of the same gender [25]. Some of the first studies focusing on the income of transgender people found that they have a lower (household) income than cisgender individuals [26,27]. Research shows that female-to-male transition is associated with a slight increase in earnings, whereas male-to-female transition results in almost one-third lower earnings [28]. In addition, there is evidence of an association between sexual orientation and leadership positions. Although most studies have found that gay men hold management or high-level management positions less often than heterosexual men, the results for lesbian women are mixed [29–33]. Furthermore, in most experimental studies, LGBTQ* people experienced more hiring discrimination and were less likely to be invited to job interviews than cisgender heterosexual people [8,34]. These results dovetail with the high share of LGBTQ* people who report experiencing discrimination in the workplace [1,6; for Germany, see 7].

Discrimination can have extensive consequences for health, well-being and behavior. Several studies based on the minority stress model [35,36] have found evidence that discriminatory experiences influence the mental health, and well-being of LGBTQ* people and that LGBTQ* people differ from cisgender heterosexual people in both respects [for Germany, see 37–39]. However, studies on the job satisfaction of LGBTQ* people have found that LGBTQ* people have lower job satisfaction than cisgender heterosexual people [40]. However, theoretical approaches and first empirical results suggest that LGBTQ* people’s career outcomes are affected not only by the direct effects of discrimination (seen, for instance, in employer hiring decisions, wage and salary levels) but also by indirect effects of discrimination, whereby prior discriminatory experiences and the goal of avoiding future discrimination shape LGBTQ* people’s career interests and decisions [41].

Based on coping theories, it is assumed that (anticipated) discrimination causes psychological imbalance and discriminated groups use coping strategies to rebuild their psychological balance [18,21,22]. Minority groups can use different strategies to deal with experienced or anticipated discrimination. Recent research has discussed a variety of strategies, such as applying for a wider range of jobs to increase the available job options [21], choosing specific occupations (such as self-employment) to avoid future discrimination [42], or choosing a safe and accepting work environment that will protect them from discrimination [19], also referred to as a “safe haven” [41]. In line with these theoretical assumptions, several studies have found that LGBTQ* people work in different occupations and industry sectors than cisgender heterosexual people [7,14,16]. Moreover, Plug et al. [15] found, based on data from the Australian twin registers, that gay men and lesbian women shy away from prejudiced occupations, and concluded that the sexual prejudices of employers play an important role in gay and lesbian workers’ occupational choices. Additional studies report that LGBTQ* people differ from cisgender heterosexual people in their occupational interests and work values [17–19]. Studies on the representation of sexual minority people in science, technology, engineering, and mathematics (STEM) fields, however, show differences by sexual orientation and also discuss the effects of discrimination and the social climate in STEM fields [43,44].

### Avoidance of discrimination and job preferences among LGBTQ* people

The decision for or against a job is driven, among other factors, by individual preferences for particular job attributes. Over the long term, these decisions can affect career trajectories. Konrad et al. [45] define the preferences for job attributes as qualities and outcomes that prospective employees desire and would expect from their work. The job attributes in question could include earnings, job security, and career opportunities. In the context of inequality, recent research has shown that job attribute preferences differ by inequality dimensions (e.g., race) [46,47], and some experimental studies have also shown differences by gender [48,49].

However, first empirical results on the job attribute preferences of LGBTQ* people show that they value not only interesting work and safe and healthy working conditions but also an LGBTQ*-friendly climate [7,50]. In the decades since the women’s and civil rights movements of the 1960s and the more recent gay rights movement, the term “diversity” has become ubiquitous in the labor market, and an increasing number of employers have made diversity management part of their company policies. Empirical results show that diversity management and equal opportunity in the workplace can increase the productivity of a company, the well-being of its LGBTQ* employees and reduce discrimination [51–55]. However, evidence on the relative value of an LGBTQ*-friendly climate and diversity management compared to other job attributes is largely missing. Our study focuses on five different job attributes: 1) income, 2) overtime, 3) promotion prospects, 4) LGBTQ*-friendly work climate, and 5) diversity management.

In line with previous research, this study hypothesizes that job attributes differ in their relative importance for LGBTQ* people. Based on empirical results of labor market discrimination against LGBTQ* people and theoretical assumptions about the connection between (anticipated) discrimination and coping, we assume that an LGBTQ*friendly work climate and diversity management are crucial job attributes for LGBTQ* people compared to other job attributes.

### Group-specific differences within the LGBTQ* community

The concept of hegemonic heteronormativity, which expands previous queer theories, states that the heteronormative binary of ‘normative’ and ‘deviant’ sexualities and genders is dynamic and evolvable and allows a shift of hegemonic power to groups that were previously seen as ‘deviant’ [56,57]. Based on this, Allen & Mendez [56] assume that some parts of the LGBTQ* community (e.g., gay men and lesbian women) are more privileged than others (e.g., bisexual people). Regarding (labor market) discrimination, some studies found differences between lesbian, gay, and bisexual individuals but the results are mixed [e.g.,^1^,^58^]. Based on previous research and the concept of hegemonic heteronormativity, we assume that lesbian, gay, and bisexual people may differ in their relative importance of job attributes caused by the assumption of different levels of discrimination and disadvantages in the society and the labor market.

However, previous research found that gender minority people experience higher levels of discrimination compared to sexual minority people [1,6,7]. This is in line with lower levels of social acceptance and legal equality [1] and is also reflected in poorer labor market and health outcomes [1,11,38]. Finally, we hypothesize differences in the relative importance of job preferences by gender identity and assume that an LGBTQ*-friendly work climate and diversity management are particularly important for gender minority people compared to cisgender people of the LGBTQ* community. Moreover, in line with previous research about differences in job preferences between cisgender men and cisgender women (regardless of sexual orientation), we assume that income and promotion prospects have a higher relative importance for cisgender men of the LGBTQ* community than for cisgender women of the LGBTQ* community [e.g., 49,59,60]. Moreover, we expect that an LGBTQ*-friendly work climate and diversity management have a higher relative importance for cisgender women of the LGBTQ* community, which would be in line with previous research that found that the general work climate is more important for cisgender women than for cisgender men [60].

## Data and methods

### Data collection

The data for our study were collected in 2021 as part of the “LGBielefeld 2021” online sample recruited through social media [61]. Using advertisements on the social network Facebook, lesbian, gay, bisexual, intersex, transgender, queer, and other non-cisgender and non-heterosexual people were recruited in an online survey on the living conditions of LGBTQ* people in Germany. Participation in the survey was limited to people 18 years of age and older. The opening screen of the survey stated that participation was confidential and voluntary and included a link for more information about the legal rights of survey participants regarding their personal data. By clicking on a “next” button, participants had to consent to process their data, state that they agree with the conditions of the survey, and participate voluntarily. LGBielefeld data collection was approved by the Bielefeld University Ethics Committee. The survey was conducted between September 3 and October 1, 2021, and resulted in 7,607 completed interviews. As a result of the sampling procedure, the data were based on non-probability selection. Given the paucity of data on LGBTQ* people in Germany and the significant sampling limitations of smaller subgroups of this generally rare target population, the data offer valuable insights into an otherwise often ignored segment of Germany’s population. A detailed description of the data, online recruitment, and materials used in the survey can be found in the accompanying data report [61].

### Sample selection

The study sample was further narrowed for the present analysis. We included only people who could be uniquely identified as belonging to the LGBTQ* population, that is, having a non-heterosexual sexual orientation and/or being transgender or non-binary or having another non-cisgender gender identity. People who were cisgender and heterosexual were excluded (N = 84), as were interviews for which sexual orientation or gender information was not available (N = 152). Based on the assumption that the decision for or against an employer is particularly relevant for the general working-age population, the sample was further reduced to individuals between the ages of 25 and 54. We thus excluded people who were either starting their careers or close to retirement (N = 1,581), as we assume that job preferences and decisions regarding employers are less relevant for them. We also excluded individuals in self-employment (N = 466), for whom we also assume that job descriptions and employer characteristics are less relevant. Finally, we excluded people for whom no further information on their occupational status was available (N = 152) or who were unemployed (N = 626). The following analyses consider the responses of 4,507 participants.

Table 1 shows the summary statistics for relevant variables describing the sample composition.

**Table 1:** Characteristics of participants in the LGBielefeld 2021 study.

	Obs.	Mean	SD	Min.	Max.
Age	4,507	35.80	7.75	25	54
Children in household (1 = yes)	4,482	0.14	-	0	1
Partner (1 = yes)	4,465	0.75	-	0	1
East Germany (1 = yes)	4,503	0.11	-	0	1
University degree (1 = yes)	4,507	0.47	-	0	1
Gross income	3,495	3,579.63	1,862.27	200	15,850
Net income	3,964	2,337.36	1,088.98	200	10,000
Contracted weekly working hours	4,019	35.73	7.97	1	80
Actual weekly working hours	3,919	38.97	11.14	1	100
General life satisfaction	4,360	7.10	2.11	0	10

Notes: Overall N = 4,507; Source: LGBielefeld 2021, own calculations.

For an overview of potential bias, we compared our analytical sample with LGBTQ* and cis-heterosexual respondents of the Socio-Economic Panel (SOEP) (S1 Table). The SOEP is one of the largest household surveys worldwide with approximately 30,000 respondents per year. A boost sample of LGBTQ* people in 2019 (SOEP-Q) increased the analytical sample of the SOEP [62]. We used the survey year 2021, set sample restrictions comparable to our analytical sample of the LGBielefeld 2021 study, and excluded respondents with no information about sexual orientation or gender identity. Overall, respondents of the LGBielefeld 2021 study are a little bit younger, higher educated, and live less often in Eastern Germany than LGBTQ* and cis-heterosexual respondents of the SOEP. LGBTQ* respondents of both studies live less often with children in a household compared to cis-heterosexual people and only LGBTQ* respondents of the SOEP are less often in a partnership compared to cis-heterosexual people of the SOEP. There are moreover small differences in income and working hours between the studies. Participants of the LGBielefeld 2021 study have a higher income and higher actual weekly working hours than LGBTQ* respondents of the SOEP (which may also be affected by differences in age).

### Measurement of sexual orientation and gender identity

Sexual orientation was measured by the sexual identity of respondents. They were asked to answer the following question: “How would you describe yourself: Are you…?,”, with seven different answer categories including an open-response format for respondents with a sexual orientation not listed (S2 Table). Open responses were coded and combined into the category “other sexual orientation”. Because there were very few cases in some categories, we focused further analyses on two categories: 1) “lesbian or gay” and 2) “bi- or pansexual” (S3 Table). The LGBielefeld 2021 study asked two questions about gender and sex (sex assigned at birth and gender identity; see S2 Table). However, we use the two-step approach to identify transgender respondents who indicated a male or female gender instead of choosing the “trans*” category [62]. Due to the relatively small number of cases in the non-cisgender categories, we combined them to arrive at three categories for gender identity, which we used in the analyses: 1) “cisgender men of the LGBTQ* community,” 2) “cisgender women of the LGBTQ* community,” and 3) “transgender, non-binary and other” (S4 Table). Table 2 gives an overview of the distribution of sexual orientation and gender identity (SOGI) for the sample population.

**Table 2:** SOGI categories of participants in the LGBielefeld 2021 study.

Sexual orientation	Gender Identity			
	Cisgender men of the LGBTQ* community	Cisgender women of the LGBTQ* community	Transgender, non-binary, and other	Total
**Lesbian or gay**	1,596 (36.82%)	1,530 (35.29%)	103 (2.38%)	3,229 (74.46%)
**Bi- or pansexual**	115 (2.65%)	580 (13.38%)	236 (5.44%)	931 (21.48%)
**Other**	7 (0.16%)	62 (1.43%)	106 (2.45%)	175 (4.04%)
**Total**	1,718 (39.63%)	2,172 (50.10%)	445 (10.27%)	4,335 (100.00%)

Notes: Overall N = 4,507; N = 172 missing information for gender identity or sexual orientation not included in the cross table; Source: LGBielefeld 2021, own calculations.

Because our sample consists of only sexual and gender minority respondents, people with a cisgender identity have a non-heterosexual sexual orientation, and people with a heterosexual orientation (included in the other category) have a non-cisgender gender identity. Based on the number of cases in these groups, it is only possible to distinguish between gender identity or sexual orientation in our analyses. A comparison of groups differentiated by sexual orientation and gender identity is not possible.

### Discrete choice experiment

Given the expected values that LGBTQ* people ascribe to job attributes, as described above, using a discrete choice experiment proves to be a powerful tool. This methodological approach, based on the understanding that individuals’ preferences vary, is critical in determining how different job characteristics are valued. It can offer a superior experimental framework to standard survey questions, especially since preference data are difficult to collect, and high bias is expected when asking certain questions directly. The basic theoretical assumption behind this model is that individuals do not have homogeneous preferences but instead choose the alternative that yields the highest individual benefit (following Lancaster’s consumer demand [63,64]). With the help of a discrete choice experiment, it is possible to approximate the utility (following McFadden’s [65] random utility theory) or the value of an attribute—in our case, a job attribute. Against this backdrop, the utility *U*_*jn*_ of an individual *n* for the alternative *j* can be assumed to be a function of its attributes:

$$U_{jn}=X_{jn}\beta_{jn}+\varepsilon_{jn}$$ (1)

where *X*_*jn*_ is a vector of attributes describing the alternative *j* for participant *n* with a set of weights *β*_*jn*_, which establish the relative contribution of each attribute to the utility associated with the alternative *j*, and *ε*_*jn*_, which is the residual capturing the unobserved variation in the characteristics of different options and any measurement errors. Several studies have used this experimental procedure to examine job preferences (e.g., to examine job decisions in the health sector [66–68], academia [69], and preferences for particular work arrangements [49]).

### Attributes and levels in the choice experiment

Respondents were asked to indicate their preferences for several alternatives in a series of choice sets in this experimental setting. A choice set consists of several alternatives that differ in various characteristics, which we refer to here as attributes. By varying the levels of attributes across alternatives and choice sets, it is possible to determine the preferences of individuals and the relative importance of specific attributes. We designed a randomized discrete choice experiment to examine the relevance of and trade-off relationships between different job attributes. This allowed us to randomly vary the characteristics of job descriptions.

Our study focuses on five different job attributes: 1) income, 2) overtime, 3) promotion prospects, 4) LGBTQ*-friendly work climate, and 5) diversity management. We consider income, overtime, and promotion prospects as common job characteristics that influence individual job decisions. Recent research shows differences in these outcomes by sexual orientation and gender identity [11,25]. We additionally use an LGBTQ*-friendly work climate and diversity management to measure two dimensions of nondiscriminatory working environments. Diversity management can include for example workshops and training on diversity, promotion of LGBTQ* networks in the company, counseling and support for employees who have experienced discrimination, and is defined as a strategy of employers to increase diversity and equality at a company. With an LGBTQ*-friendly work climate, we address the actual situation at the workplace independent of the presence of diversity management. Previous research measures the work climate for LGBTQ* people based on the extent of support and hostility against this group (e.g., if LGBTQ* employees are treated with respect, if LGBTQ* employees feel accepted by coworkers, and if LGBTQ* employees must be secretive) [52]. Based on this, we assume that an LGBTQ*-friendly work climate is, next to the protection of LGBTQ* employees against hostility, also characterized by support of LGBTQ* employees. According to recent research, diversity management and equal opportunity in the workplace can help LGBTQ* people to come out at work, increase their mental health and well-being, and reduce discrimination [51,53–55].

Before starting our study, we pretested the online survey, including the choice experiment. This allowed us to address technical issues and check respondents’ presumptions about the wording of our experimental design. We showed all respondents in the pretest (N = 55) five fictional job descriptions and then integrated five questions with open-response fields. The results of the pretest show that not all respondents understand the term “diversity management.” We, therefore, decided to add some examples of diversity management to our experimental design (see S1 Fig). Furthermore, our first version of the experimental design included a sixth job attribute (openness of coworkers regarding sexual orientation and gender identity). However, the pretest showed that not all respondents could differentiate between an LGBTQ*-friendly work climate and the openness of coworkers regarding sexual orientation and gender identity, so we decided not to use this attribute in our final experimental design. Overall, for most respondents, the understanding of an LGBTQ*-friendly work climate is in line with our definition as a workplace that offers protection of LGBTQ* employees against hostility and support of LGBTQ* employees (typical answers were for example “No hostility, openness, understanding, tolerance and respect” or “That no one is discriminated against and everyone has the same opportunities for advancement”). Moreover, some respondents highlight especially the importance of equal treatment (e.g., “Treat them like anyone else” or “Just a normal way of dealing with sexualities outside heterosexuality”).

In our final discrete choice experiment, participants had to choose between two hypothetical job descriptions (job A and job B) and an opt-out option “neither” in six choice sets. Each job was described by the five attributes (see Table 3). The opt-out option was included to increase the external validity of the discrete choice data [70]. In the experimental setting, the different attributes varied in their expression. Respondents were offered a choice set, in which they chose the stimulus, that is, the job description, they preferred. Based on the theoretical background of this study, it can be assumed that participants select the job description that offers or appears to offer them the highest overall utility. In the questionnaire, we mentioned that the descriptions were hypothetical job descriptions and that each description contained information about working conditions. Additionally, participants were asked to disregard their current job situation and assume that all the characteristics not mentioned were the same for all job descriptions. S1 Fig presents an example of the choices that survey participants were given. Based on the participants’ choices, we were able to assess their preferences for the job conditions.

**Table 3:** Attributes and levels.

	Job attribute	Levels
1	Gross income (per month)	3,000 € 3,500 € 4,000 € 4,500 € 5,000 €
2	Overtime (per month)	0 hours 2 hours 6 hours
3	Promotion prospects	After 3 years After 4 years After 5 years
4	Diversity management	Yes No
5	LGBTQ*-friendly work climate	Yes No

### Experimental design

The number of possible attribute combinations with two two-level attributes, two three-level attributes, and one five-level attribute is 180 (2^2^*x*3^2^*x*5^1^). Each choice question offers two options, leading to a total of 32,220 possible choices (180*x*179). As this is an extremely large number of choices, it was necessary to select a manageable number of meaningful choice sets. To reduce the number of choices, we applied a D-efficient sampling strategy to select a fraction of the choice sets. We used the Stata ado *dcreate* [71] to sample the fractional design, maximizing the D-efficiency of the design to 4.401. The choice set was reduced to 36 scenarios, containing a total of 66 alternatives. Due to the D-efficiency-maximizing approach, 6 alternatives are repeated in the design. More detailed description of the distribution of attribute levels and the orthogonality of the model can be found in the S5–S10 Tables in the appendix. The total of 36 choice scenarios were split into six blocks to reduce the burden on each respondent. Each participant was randomly assigned to one block and asked to make six decisions.

### Field results

The survey respondents were allowed to skip the experimental task. Only two of the overall 4,507 (0.04 percent) eligible respondents decided not to make any decisions. This reduced the final sample for analysis of the choice experiment to 4,505 eligible respondents. 4,459 (98.98 percent) of the remaining respondents answered all six choice questions; 46 (1.02 percent) answered at least some choice questions (see Table 4).

**Table 4:** The number of completed choice sets per respondent.

No. completed choice sets	Obs.	%	Cum. %
1	1	0.02	0.02
2	1	0.02	0.04
3	6	0.13	0.18
4	11	0.24	0.42
5	27	0.60	1.02
6	4,459	98.98	100.00
**Total**	**4,505**	**100.00**	

Source: LGBielefeld 2021, own calculations.

Furthermore, 82 respondents (1.8 percent) chose the “neither” option to opt out of all six questions. However, 2,647 (58.8 percent) did not opt out of any of the questions, indicating that, in most cases, participants displayed clear tendencies (S2 Fig).

## Analysis

Traditionally, discrete choice experiments are analyzed using logistic regression models, such as multinomial and conditional logistic regression [65]. Although these models are widely used, they have limitations. For example, they do not allow for possible respondent heterogeneity unless these are observable variables. In addition, these models assume independence of irrelevant alternatives, which can lead to unrealistic predictions. Because of these drawbacks, mixed logit models (also known as random parameter logit models) are being used to an increasing degree. These models allow for variation in preferences for specific attribute values and improve the results. Furthermore, a mixed logit model also allows for modeling of repeated choices by the same individual, as in the present study [72,73].

In a choice experiment, participants *N* (*n* = 1,…,*N*) must choose between job descriptions *J* (*j* = 1,…,*J*) in each of the *T* choice sets (*t* = 1,…,*T*). According to random utility theory, the utility that a participant *n* receives from a job description *j* in a choice set *t* can therefore be described as follows:

$$U_{jnt}=\beta_{0}+\beta_{jn}X_{jnt}+\varepsilon_{jnt}$$ (2)

Here, *β*_0_ refers to the alternative specific constants and *X*_*jnt*_ is a vector of observed explanatory variables related to the alternative *j* and respondent *n*. *β*_*jn*_ is a vector of individual-specific parameters associated with the observed variables, including the alternative specific constants. The parameters *β*_*jn*_ vary across individuals and are assumed to be normally distributed with density function *f*(*β*|Θ), where Θ refers to the mean and the covariance of *β*.

Moreover, *ε*_*jnt*_ is an unobserved random term that varies over the participants *N*, the job descriptions *J*, and the choice sets *T*. It is assumed to follow a Gumbel distribution, with a variance given by $Var(\varepsilon_{jnt})=\mu_{n}^{2}(\pi^{2}/6)$, where *μ*_*n*_ is an individual-specific scale parameter for participant *n*. This scale parameter *μ*_*n*_ represents the variability of the unobserved portion of utility across individuals *N*. Normalizing the variance of the unobserved portion of utility leads to a normalized random term that is independently and identically distributed [65] over participants *N*, job descriptions *J*, and choice sets *T* with a variance equal to (*π*^2^/6).

In our model, respondents are confronted with three choices involving job descriptions: Two specific job alternatives with five attributes (*I* income, *O* overtime, *P* promotion prospects, *D* diversity management, *C* work climate) in each choice task and an opt-out option that represents a “neither” choice. The utility derived by participant *n* from each alternative in each choice task can be expressed as follows:

$$\mathrm{Job}\;A:\;U_{n1t}=\beta_{n1}I_{n1t}+\beta_{n2}O_{n1t}+\beta_{n3}P_{n1t}+\beta_{n4}D_{n1t}+\beta_{n5}C_{n1t}+\varepsilon_{n1t}$$ (3)

$$\mathrm{Job}\;B:\;U_{n2t}=\beta_{n1}I_{n2t}+\beta_{n2}O_{n2t}+\beta_{n3}P_{n2t}+\beta_{n4}D_{n2t}+\beta_{n5}C_{n2t}+\varepsilon_{n2t}$$ (4)

$$\mathrm{Opt}‐\mathrm{out}:\;U_{n3t}=\beta_{0}+\beta_{n1}I_{n3t}+\beta_{n2}O_{n3t}+\beta_{n3}P_{n3t}+\beta_{n4}D_{n3t}+\beta_{n5}C_{n3t}+\varepsilon_{n3t}$$ (5)

Based on the theoretical assumptions, it can be assumed that participant *n* selects the job description *j* that provides the highest utility *U* from a choice set *t*. Therefore, the probabilities of choosing job A over job B and job A over the opt-out option can be described as:

$$Prob(U_{n1t}-U_{n2t}>0)$$ (6)

$$\mathrm{and}\;Prob(U_{n1t}-U_{n3t}>0).$$ (7)

These probabilities are calculated based on the utility differences between alternatives using the utility expressions and the assumption of random utility maximization.

We employed an initial analysis using a more traditional conditional logit model as a benchmark model and a mixed logit model following Revelt and Train [73] as an alternative model specification. Goodness-of-fit criteria, that is, Akaike and Bayesian information criteria, were used to determine the best model for our choice data. To examine how job attribute preferences differ based on gender identity and sexual orientation, we estimated separate mixed logit models for each sexual orientation and gender identity (SOGI) group. For sexual orientation, we present results for the ‘lesbian or gay’ and ‘bi-or pansexual’ categories, as the sample size for the ‘other’ category was too small for reliable analysis.

We estimated mixed logit models with the income, overtime, and promotion prospect attributes as fixed, as we assumed that all individuals have the same preference for a higher salary, less overtime, and fewer years until promotion. The remaining attributes were treated as random. We used effect coding, which assigns a unique coefficient for each attribute level, ensuring that the effects are not directly correlated with the intercept [74]. To account for the block to which respondents were assigned, we included interactions with an alternative-specific constant identifying the opt-out alternatives. All analyses were performed in Stata 17.0. Mixed logit models were estimated using the user-written Stata command *mixlogit* [75]. Since each respondent made up to six choice decisions, we did not rely on independent observations. To take this into account, we computed individual clustered standard errors. We computed all mixed logit models with 500 Halton draws to approximate the log-likelihood function [76,77].

Additionally, we calculated predicted probabilities to assess the potential effect of each attribute level on the decision for a hypothetical job description. By comparing models with one attribute level changed while the others remain constant, the specific effect of an attribute level on predictions can be isolated. The average effect of an attribute level (${\bar{E}}_{ik}$) on a decision is given by:

$${\bar{E}}_{ik}=\frac{1}{N}\sum_{n=1}^{N}(\bar{P_{n_{jk}}}-\bar{P_{n_{jr}}})$$ (8)

Here, *N* is the sample size, $\bar{P_{n_{jk}}}$ is the individual average predicted probability for the decision when the attribute *i* of alternative *j* is set to level *k*, and $\bar{P_{n_{jr}}}$ is the predicted probability when the same attribute *i* is set to a reference level *r*. The user-written Stata command *mixlpred* was used for this purpose [75].

Using estimates from the mixed models, we also determined the relative importance of job characteristics in explaining decisions about a hypothetical job description. The relative importance (*RI*_*i*_) of each attribute, that is, the importance of an attribute relative to all other attributes, was calculated as the difference in preference weights between the most and least preferred levels of the same attribute [74]:

$$RI_{i}=\frac{\max\left(\beta_{ij}\right)-\min(\beta_{ij})}{\sum_{i=1}^{I}\left(\max(\beta_{ij}\right)-\min(\beta_{kji}))}$$ (9)

Where *I* represents the total number of attributes in a choice experiment, max(*β*_*ij*_) denotes the maximum part-worth utility associated with the *j*-th level of an attribute *i*, while min(*β*_*ij*_) represents the minimum part-worth utility linked to the level *j* of attribute *i*. For this computation, we resorted to the user-written Stata command *mixlbeta* [75,78].

## Results

### Preferences for job attributes in general

To address our main research question, we initially explore job preferences within the entire LGBTQ* community. The estimates of preference weights for the effects-coded conditional logit and mixed logit models are shown in Table 5. We observed a substantial difference in goodness of fit between the two models. The log-likelihood value, the Akaike information criterion (AIC), and the Bayesian information criterion (BIC) indicate that the mixed logit model, which assumes heterogeneity in preferences, provides a better goodness of fit for our choice data. The log-likelihood value improved from -19754.82 in the conditional logit model to -16544.94 in the mixed logit model. The AIC improved from 39541.65 to 33137.88, and the BIC from 39690.45 to 33361.1. However, the different specifications provide nearly similar results. When comparing the two models, we found that the estimated coefficients in the mixed logit model tend to be larger. This can be attributed to the accounting for variation in individuals’ preferences. The significant between-subject standard deviations (SD) in this model further amplify the presence of variation in preferences.

**Table 5:** Results of the model comparison.

	CL		MXL	
	Coef.	SE	Coef.	SE
**Main**				
Income				
3,000 €^1^	-0.893		-1.551	
3,500 €	-0.575***	0.021	-0.870***	0.035
4,000 €	0.221***	0.024	0.444***	0.035
4,500 €	0.424***	0.023	0.688***	0.037
5,000 €	0.823***	0.022	1.289***	0.038
Overtime				
0 hours^1^	0.487		0.700	
2 hours	0.165***	0.014	0.301***	0.022
6 hours	-0.652***	0.023	-1.001***	0.038
Promotion prospects				
3 years^1^	0.061		-0.015	
4 years	0.131***	0.017	0.250***	0.027
5 years	-0.192***	0.018	-0.235***	0.027
Diversity management	0.295***	0.011	0.499***	0.018
Work climate	1.017***	0.016	1.655***	0.036
**SD**				
Diversity Management			-0.380***	0.030
Work Climate			1.020***	0.027
Log-likelihood (full model)	-19754.82		-16544.94	
Prob. > chi2	0.0000		0.0000	
AIC	39541.65		33137.88	
BIC	39690.45		33361.1	
Respondents	4505		4505	
Job descriptions	80862		80862	

Significance levels

* p<0.05

** p<0.01

*** p<0.001

^1^ Reference value; Note: The estimated coefficients in column CL are from the conditional logit (CL) model that assumes homogenous preferences for individuals. The estimated coefficients in column MXL are from the mixed logit model (MXL) that accounts for the individual heterogeneity. In the MXL, all attributes except income, overtime, and promotion are random. Results for complete models can be found in S3 Table; Source: LGBielefeld 2021; own calculations.

Estimated effects-coefficients represent the relative contribution of an attribute level to the utility assigned by respondents to an alternative. Overall, the estimated results indicate that respondents prefer a workplace with high income, low overtime, fewer years to promotion, the presence of diversity management at the company level, and an LGBTQ*-friendly work climate. All coefficients align with the expected direction, except promotion prospects, which exhibit a negative coefficient for the first and third categories and a positive coefficient for the second category. We assume that the small distance between the categories (1 year) makes it difficult for respondents to factor this in their hypothetical job decisions. This observation aligns with the relatively low relative importance of promotion prospects (see Fig 2).

**Fig 1:**
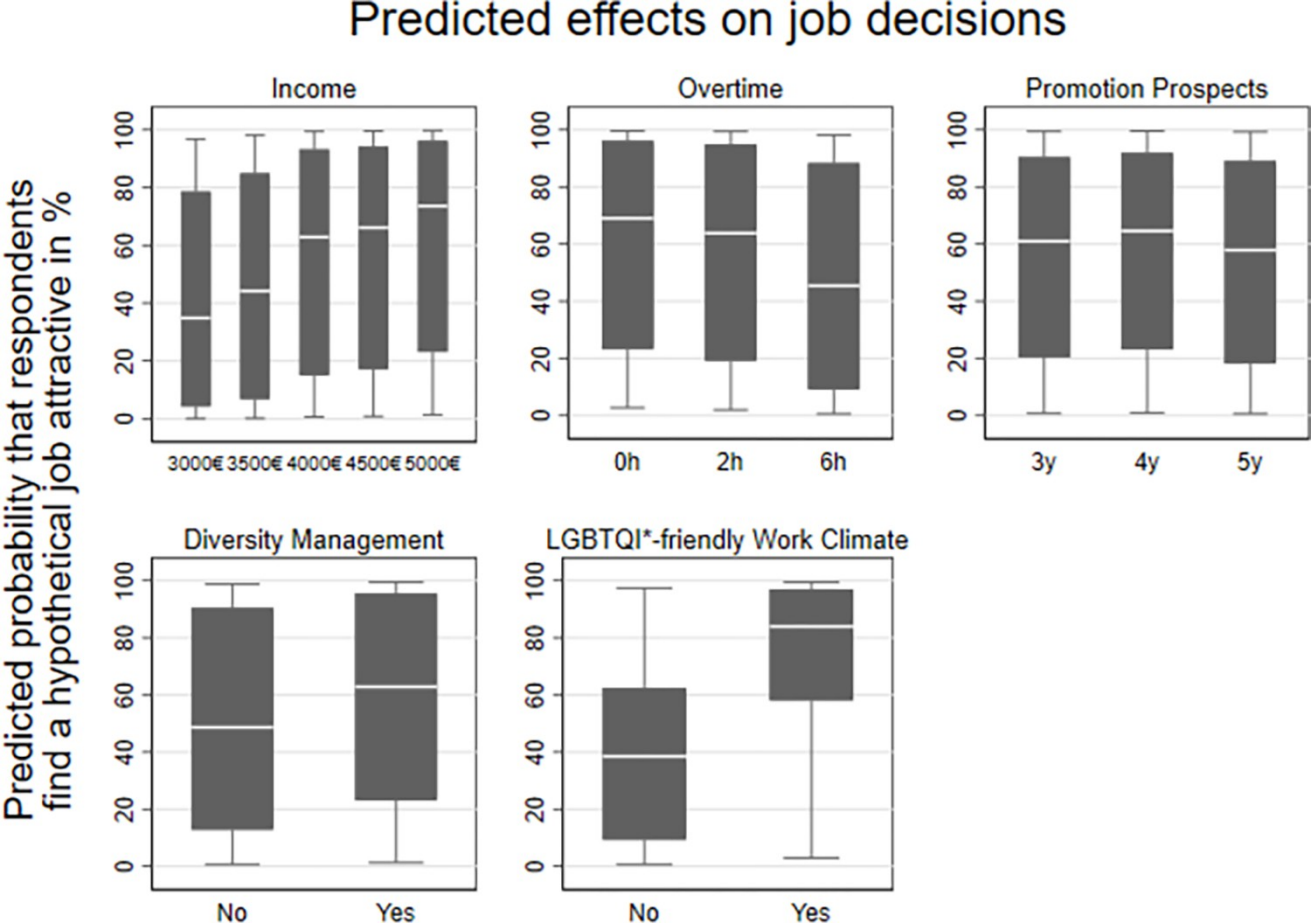
Predicted effects on job decisions. Note: Pooled data, N = 80,862; Source: LGBielefeld 2021; own calculations.

To further explore the relative contribution of an attribute level on the utility assigned to an alternative, Fig 1 offers a comprehensive insight into the anticipated impact of different attribute levels on respondents in finding a hypothetical job attractive. Utilizing preference estimates derived from the mixed logit models, we predicted the probability of participants selecting a particular job description at different attribute levels given. The boxplots within each graph illustrate the distribution of these predicted probabilities among respondents. Higher-placed boxplots suggest greater predicted probabilities associated with specific attribute levels.

The predicted probability of respondents choosing a hypothetical job increased from 34.8 percent when the job offered an income of 3000 euros per month to 73.6 percent with an income of 5000 euros per month. On the other hand, the predicted probability decreased when more overtime was involved, going from 69.0 percent (0 hours per month) to 45.4 percent (6 hours per month). Interestingly, better promotion prospects have a mixed effect, with the predicted probability increasing from 61.0 percent (3 years to wait for a promotion) to 57.9 percent (5 years to wait for a promotion). Additionally, the presence of diversity management and an LGBTQ*-friendly work climate had a positive impact on the predicted probability of respondents choosing a hypothetical job. Specifically, the predicted probability increased from 48.7 to 62.8 percent when diversity management was present, and from 38.5 to 83.9 percent when an LGBTQ*-friendly work climate was mentioned.

**Fig 2:**
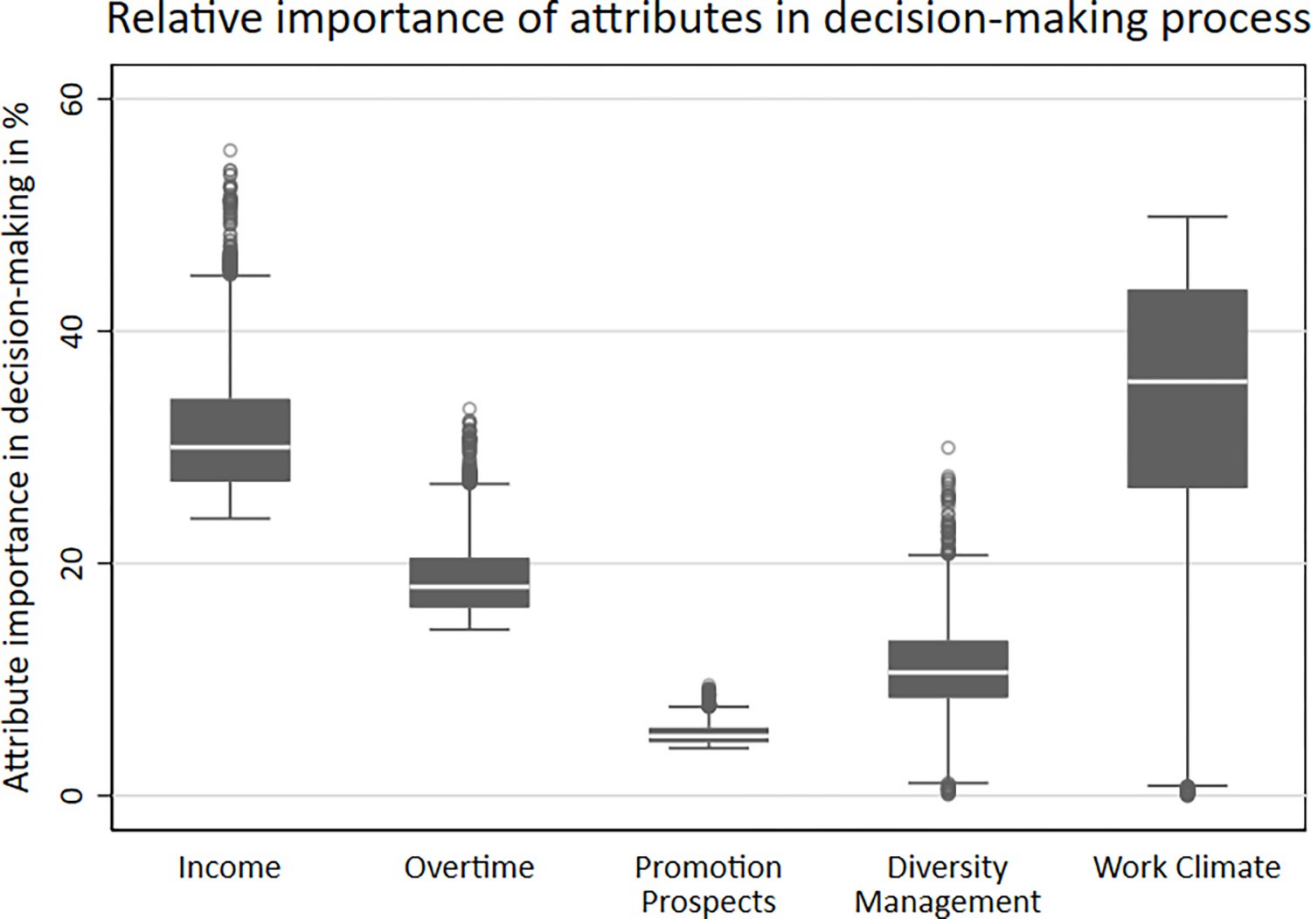
The relative importance of attributes in the decision-making process. Note: Pooled data, N = 80,862; Source: LGBielefeld 2021; own calculations.

Fig 2 illustrates the relative importance of individual job attributes in the decision-making process, as determined by a mixed logit model with effects-coding. The graph presents five boxplots, each representing the average importance of a specific job attribute relative to the other attributes. The positions of these boxplots offer insights into the hierarchical importance of these attributes in decision-making. Higher-placed boxplots indicate attributes with greater relative importance, while lower-placed ones signify attributes of lesser importance. The results indicate that nearly two-thirds of the relative importance in respondents’ decisions can be attributed to an LGBTQ*-friendly work climate and income. Specifically, the LGBTQ*-friendly work climate accounted for an average of 33.8 percent of respondents’ decisions, while the presence of diversity management accounted for 11.0 percent. Among the traditional job characteristics, income played a particularly important role in decision-making, accounting for 31.2 percent. The amount of overtime contributed 18.7 percent to the decision-making process, whereas promotion prospects held relatively lower importance at 5.3 percent. In line with our initial hypothesis, job attributes differ in their relative importance for LGBTQ* people. Whereas an LGBTQ*-friendly work climate is a crucial job attribute, diversity management is less important for LGBTQ* people.

### Group-specific preferences for job attributes

We conducted models by sexual orientation and gender identity to examine differences within the LGBTQ* community. S12 and S13 Tables display the results of the mixed logit models for each sexual orientation and gender identity group included in the study. Overall, the estimates confirm the general tendencies observed across the different groups. While the model coefficients cannot be compared directly, as they are confounded with a scale parameter that may differ between groups, the relative distances between the attribute levels exhibit minimal variations across groups, indicating consistency in the overall patterns.

**Fig 3:**
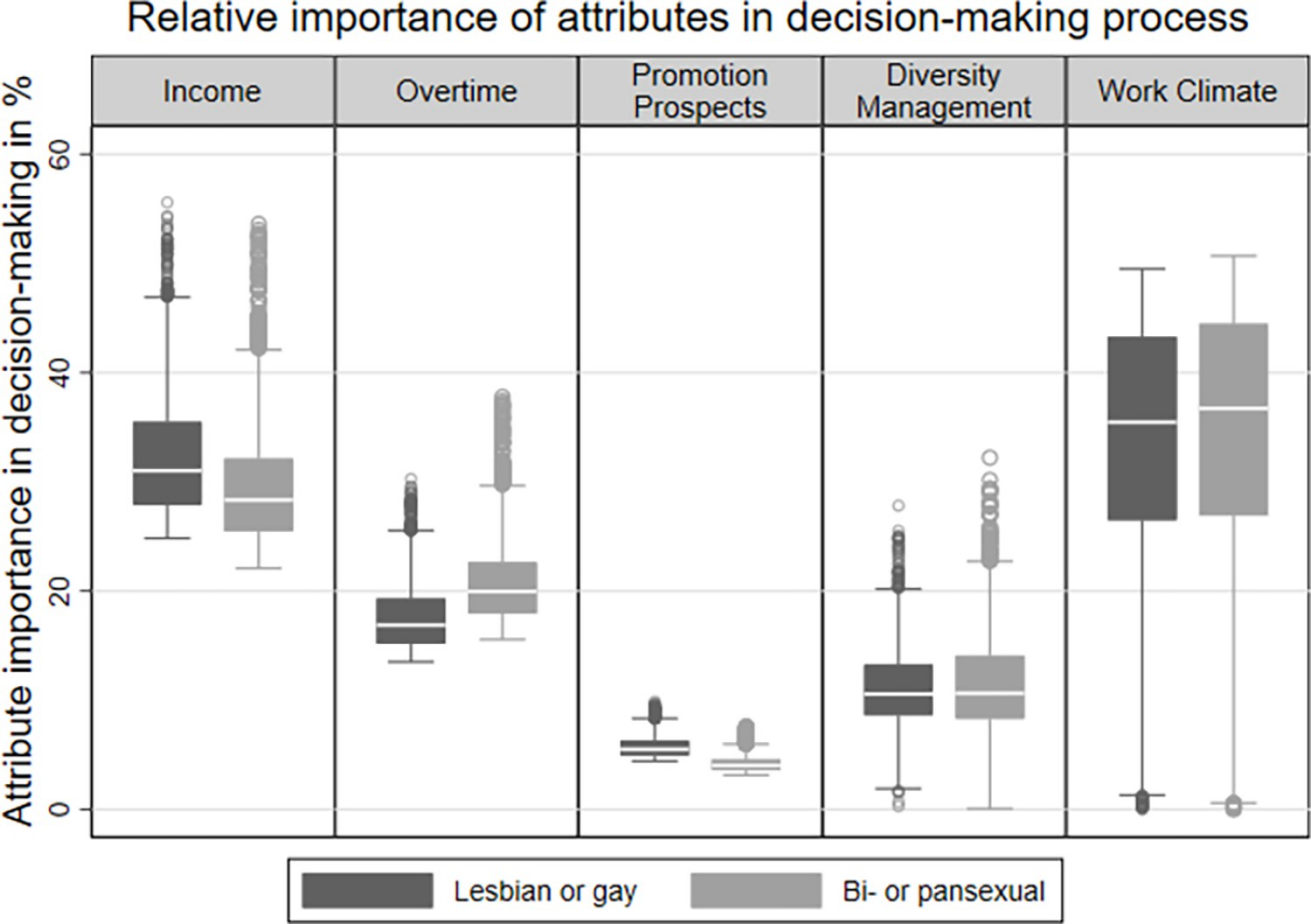
The relative importance of job attributes in the decision-making process by sexual orientation. Note: Pooled data, lesbian or gay: N = 3,273, bi- or pansexual: N = 1,027; Source: LGBielefeld 2021; own calculations.

To provide deeper insights into the effect of sexual orientation, Fig 3 illustrates the relative importance of each attribute in the decision-making process based on sexual orientation. When comparing the relative importance of attributes across all sexual orientations, an LGBTQ*-friendly work climate emerges as the most important factor in selecting a hypothetical job description, followed by income, unpaid overtime, and diversity management. The prospect of promotion plays the least important role in decision-making. Notably, there are only marginal differences between lesbian or gay and bi- or pansexual respondents, which is against our assumption based on previous research. Within the dimensions of diversity management and work climate, the variances are minimal, with differences below one percentage point. However, income exhibits a 2.7 percentage point higher average relative importance for lesbian or gay people, while promotion prospects have a 1.5 percentage points higher value. On the other hand, the dimension of overtime holds a 3.3 percentage points higher average relative importance for the bi- or pansexual group.

**Fig 4:**
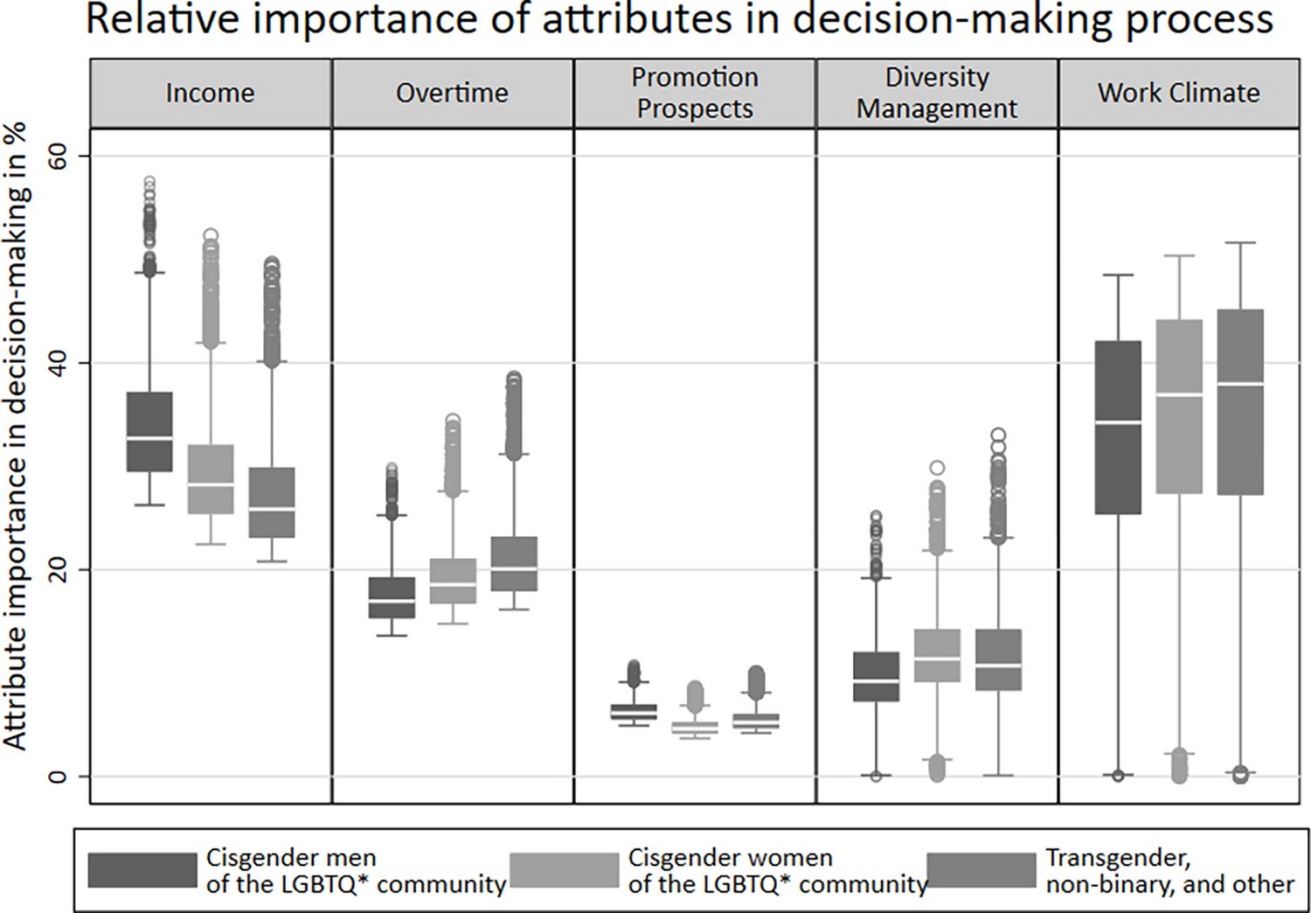
The relative importance of job attributes in the decision-making process by gender identity. Note: Pooled data, cisgender men of the LGBTQ* community: N = 1,171, cisgender women of the LGBTQ* community: N = 2,171, transgender, non-binary, and other: N = 464; Source: LGBielefeld 2021; own calculations.

Fig 4 provides a visualization of the relative importance of each attribute in the decision-making process based on gender identity. A direct comparison of the average relative importance of individual attributes reveals a similar hierarchy as observed in the analysis by sexual orientation: Regardless of gender identity, an LGBTQ*-friendly work climate emerges as the most important factor in selecting a hypothetical job description, followed by income, unpaid overtime, and diversity management. The prospect of promotion plays the least important role in decision-making.

However, differences can be observed between gender identity groups. While cisgender men of the LGBTQ* community assign approximately equal importance to income and an LGBTQ*-friendly work climate, each accounting for about 33 percent (income: 33.1%; LGBTQ*-friendly work climate: 32.5%), the other gender identity groups clearly prioritize the LGBTQ*-friendly work climate. For cisgender women of the LGBTQ* community and transgender, non-binary, and people with another gender identity, income contributes to the decision-making process at less than 30 percent on average (cisgender women of the LGBTQ* community: 29.4%; transgender, non-binary, and other: 27.2%). In contrast, an LGBTQ*-friendly work climate comprises approximately 35 percent of the decision process (cisgender women of the LGBTQ* community: 34.6%; transgender, non-binary, and other: 34.8%). Diversity management exhibits an average relative importance around two percentage points higher for cisgender women of the LGBTQ* community and transgender, non-binary, and people with another gender identity compared to cisgender men of the LGBTQ* community. Additionally, there is a slightly weaker effect of overtime in the decision-making process of cisgender men of the LGBTQ* community compared to the other groups, while there is a slightly stronger effect of promotion prospects for cisgender men of the LGBTQ* community compared to cisgender women of the LGBTQ* community and transgender, non-binary, and people with another gender identity. These differences by gender identity are with our expectations based on previous research.

### Robustness checks

To further strengthen our analysis, we conducted several robustness checks. First, we examined whether respondents who reported experiencing discrimination based on their sexual orientation or gender identity differed in their attribute preferences compared to respondents who had not experienced discrimination. We hypothesized that individuals who had experienced discrimination would place higher importance on an LGBTQ*-friendly work climate and diversity management. S14 Table presents the results of the associated mixed logit model, and S3 Fig illustrates the relative importance of job attributes in the decision-making process based on discriminatory experiences. The effects observed in both models demonstrate a consistent tendency, with individuals who experienced discrimination attributing slightly greater benefits to diversity management and an LGBTQ*-friendly work climate. Specifically, an LGBTQ*-friendly work climate exhibits a 0.8 percentage point higher average relative importance, and diversity management shows a 3.1 percentage point higher average relative importance for respondents with discriminatory experiences. However, the income displays a 5.0 percentage points lower average relative importance for respondents with discriminatory experiences. Minimal differences were found for promotion prospects and overtime. Importantly, the overall pattern of attribute preferences remains largely unchanged, indicating a modest impact of discriminatory experiences on the relative job attribute importance.

As an additional check, and to justify focusing on the primary working age (25–54 years) in our analysis sample, we examined different age groups. In S4 Fig and S10 Table, we compared the relative importance of job attributes for a younger (18 to 24 years) and older (55 to 69 years) age groups to the age group of our analytical sample (25–54 years). The attribute hierarchy remains consistent across different age groups, with an LGBTQ*-friendly work climate and income being most important for decision-making. Notably, income was assigned lower relative importance by younger and older individuals, averaging 28.9 percent for 18–24-year-olds and 24.1 percent for 55-69-year-olds, both below the main analysis sample average (31.2 percent). Conversely, diversity management and LGBTQ*-friendly work climate showed a reversed relationship. The 55–69 age group assigned a 4.0 percentage points higher relative importance to an LGBTQ*-friendly work climate, and both younger and older age groups assigned higher relative importance to diversity management (18–24 years: +1.6%-points; 55–69 years: +3.5%-points) than the main analysis population.

Further, we explored differences in job attribute importance based on occupational status, as we excluded unemployed and self-employed individuals from our analysis sample. A comparison of these occupational groups with the main analysis sample revealed the same relative hierarchy between job attributes (see S5 Fig and S16 Table) but smaller differences in the relative importance assigned by the different occupational statuses. The unemployed and the self-employed assigned less importance to income (unemployed: -4.0%-points; self-employed: -1.4%-points) and the amount of overtime hours (unemployed: -2.5%-points; self-employed: -2.3%-points) than the main analysis population. On the other hand, an LGBTQ*-friendly work climate was particularly important for the excluded group (unemployed: +4.4%-points; self-employed: +4.7%-points).

The results of the two controls for the restriction criteria, i.e. both for the age groups and for the occupational status, confirm the assumptions made previously, which led to the specific selection of our analysis sample. In both cases, although the general hierarchy between the attributes was the same, some, admittedly smaller, differences in the evaluation of the attributes could be identified. The effects found indicate moderate influences of age groups and occupational status on the relative job attribute importance.

In order to capture some sources of heterogeneity in the decision process while accounting for unobserved heterogeneity, we included interactions of key participant characteristics with the random parameters in the utility calculation. These attributes included age groups, the presence of children in a household, being in a partnership, living in East Germany, having a university degree, the net income as well as the contracted weekly working hours. S17 Table presents the results of the associated mixed logit model including the interactions. Only the interactions between net income and diversity management respectively an LGBTQ*-friendly work climate were significant, with effect sizes close to zero. These results suggest that the effect of diversity management respectively an LGBTQ*-friendly work climate varies depending on the level of net income, although the effect is comparatively small.

In addition, we conducted an analysis to investigate whether the inclusion of a “neither” opt-out option influenced the level of benefits associated with each attribute. A comparison with a mixed logit model (S18 Table) revealed no substantial differences. Even when excluding all opt-out responses, the trends identified in the main model and the level of attributed importance for individual attributes remained consistent and stable.

To assess whether six decisions per person might constitute a significant survey burden, we conducted a comparison between the full model and a model limited to the first three decisions per person (S19 Table). While the overall patterns of attribute preferences remain consistent, there are clear indications of survey fatigue. The comparison between the two models reveals that the presumed challenge in distinguishing between the levels of the promotion prospect, as described in the Results section, does not apply to the model with only the first three decisions. This suggests that the difficulty in differentiation may be attributed to the repetitive nature of the task involving decision cards.

## Discussion

In light of ongoing discrimination faced by LGBTQ* people and their disadvantaged position in the labor market compared to cisgender heterosexual individuals, this paper aims to explore attempts of LGBTQ* people to avoid a discriminatory working environment by sacrificing other job attributes. The study specifically explores the importance of income, promotion prospects and overtime hours, LGBTQ*-friendly work climate, and diversity management in the workplace when making decisions about their job. By employing a discrete choice experiment with hypothetical job descriptions, we have gained unique insights into the link between LGBTQ* people’s job decisions and their aspirations to avoid future discrimination.

The results are in line with our hypothesis: job attributes differ in their relative importance for LGBTQ* people. More specifically, we found that an LGBTQ*-friendly work climate and income hold the highest relative importance in LGBTQ* people’s hypothetical job decisions. This underscores the significant value placed on having an LGBTQ*-friendly work environment within this population and is in line with our expectations and the theoretical background. On average, an LGBTQ*-friendly work climate accounted for 33.8 percent of respondents’ decision-making. However, diversity management only accounted for 11.0 percent and has a lower relative importance than overtime and income, which speaks against our expectation of the high importance of this attribute. Differences between the relative importance of an LGBTQ*-friendly work climate and diversity management suggest that LGBTQ* people prioritize the actual work environment more than the diversity measures implemented by employers. However, the amount of overtime contributed to 18.7 percent of the decision, while promotion prospects accounted for 5.3 percent. Considering the current state of research, these results highlight the substantial importance of an LGBTQ*-friendly work climate and the elevated value attributed to this job attribute compared to others. Even when facing discrepancies in income, which is a crucial job attribute [46], LGBTQ* people may be willing to sacrifice a higher income in exchange for the benefits of an LGBTQ*-friendly work climate.

Overall, the results show that LGBTQ* people are willing to sacrifice a high amount of career opportunities and labor market outcomes for an LGBTQ*-friendly work climate, but diversity management is less important. The findings indicate that alongside direct discrimination in the labor market, such as biased hiring decisions of employers, LGBTQ* people’s career choices driven by the intention to avoid discrimination, can impact their labor market outcomes (e.g., earnings), professional positions, and occupational segregation. These results align with recent research emphasizing the significance of an LGBTQ*-friendly work climate, the effects of discrimination on job decisions, and the coping strategies of minority groups [7,21].

While analyzing the results by gender identity, we observed that compared to cisgender men of the LGBTQ* community, both cisgender women of the LGBTQ* community and gender minority individuals assigned lower relative importance to income, while assigning higher relative importance to an LGBTQ*-friendly work climate, which is in line with our expectations based on previous research. Moreover, studies have shown that gender minorities experience higher levels of discrimination in the labor market compared to sexual minorities [7], which could explain the higher relative importance of an LGBTQ*-friendly work climate for this group. Differences between cisgender men and women of the LGBTQ* community may contribute to general differences in job preferences based on gender [46]. However, we found only minor differences based on sexual orientation, which is against our prior expectations. Further research in this field should explore the intersectionality between sexual orientation and gender identity, although detailed investigation was limited in this study due to the small number of respondents in different groups.

Furthermore, the results offer potential starting points for practical implications regarding the labor market integration of LGBTQ* people. The high relative importance of an LGBTQ*-friendly work climate should serve as motivation for employers to cultivate such an environment within their companies, contributing to the overall equality and inclusivity of LGBTQ* people. It is important for employers to recognize that simply implementing diversity management, such as equal recruitment processes or equal pay, may not be sufficient. In light of ongoing social debates surrounding diversity and the phenomenon of ‘rainbow washing’ (where companies superficially show solidarity with the LGBTQ* community for marketing purposes without genuine commitment), the preferences of marginalized groups should also be taken into account. It is crucial to genuinely involve and consider the perspectives and needs of these groups in order to create truly inclusive and supportive work environments.

While our paper makes valuable contributions to the existing research on the labor market situation of LGBTQ* people and sheds light on the understudied topic of job preferences, we acknowledge some limitations in our study: Firstly, our use of a discrete choice experiment as the method introduces the limitation that our findings are based on hypothetical decisions. Since we did not measure real job decisions, the extent to which our results are comparable to actual job decisions made by LGBTQ* people remains uncertain. However, the relative importance we observed suggests that an LGBTQ*-friendly work climate is crucial in deciding for or against a fictional job, and we assume that this attribute also plays an important role in real-life job decisions. Additionally, previous studies have demonstrated that job preferences are closely related to real-life decisions [48]. Secondly, as our sample exclusively consists of respondents from the LGBTQ* community, we are unable to examine the relative importance of job preferences of heterosexual individuals. It would be valuable to replicate the analysis with cisgender heterosexual individuals and compare their job preferences with those of LGBTQ* respondents. Thirdly, our sample was recruited through social media, which could introduce potential sampling and selection bias. A comparison of our sample with LGBTQ* and cis-heterosexual people of the SOEP shows that participants of the LGBielefeld 2021 study are a little bit younger, higher educated, and live less often in Eastern Germany. Whereas we found some differences in the number of children and partnership status, there are small differences in income and working hours between both studies. Fourthly, our experimental design includes two attributes which are not used in such designs before. Even though we pretested the understanding of both attributes in advance, it is possible that they are not clearly understandable and selective for all respondents. This applies in particular to the rather general attribute of an LGBTQ*-friendly work climate. We suggest that further research should also examine the conditions for and understanding of an LGBTQ*-friendly work climate from the community’s perspective. Lastly, it is important to consider that the LGBielefeld 2021 study included various questions about respondents’ experiences of discrimination [61]. This may have led to a priming effect, potentially influencing answers regarding the importance of an LGBTQ*-friendly work climate. However, our robustness checks indicate consistent patterns in preferences of individuals with and without discriminatory experiences.

## Supporting information

S1 Fig An example of a choice set. Notes: Translated to English.
(TIF)

S2 Fig Number of times selecting the opt-out “neither” per respondent. Notes: N = 4,505; Source: LGBielefeld 2021, own calculations.
(TIF)

S3 Fig Control–The relative importance of job attributes in the decision-making process by discriminatory experiences. Notes: Without discriminatory experiences: N = 766; With discriminatory experiences: N = 3,735; Source: LGBielefeld 2021; own calculations.
(TIF)

S4 Fig Control–The relative importance of job attributes in the decision-making process by age groups. Notes: 18–24 years: N = 459; 24–54: N = 4,505; 55–69 years: N = 293; Source: LGBielefeld 2021; own calculations.
(TIF)

S5 Fig Control–The relative importance of job attributes in the decision-making process by occupational status. Notes: Unemployed: N = 626; Employed: N = 4,505; Self-employed: N = 465; Source: LGBielefeld 2021; own calculations.
(TIF)

S1 Table Characteristics of participants in the LGBielefeld 2021 study and the Socio-Economic Panel (SOEP). Source: LGBielefeld 2021 (unweighted); SOEP v38.1 (weighted), own calculations.
(DOCX)

S2 Table Measurement of sexual orientation and gender/sex. Source: LGBielefeld 2021.
(DOCX)

S3 Table Frequencies of sexual orientation in analysis sample. Notes: Overall N = 4,507; N = 19 missing information for sexual orientation not included in table; Source: LGBielefeld 2021, own calculations.
(DOCX)

S4 Table Frequencies of gender identity in analysis sample. Notes: Overall N = 4,507; N = 153 missing information for gender identity not included in table; Source: LGBielefeld 2021, own calculations.
(DOCX)

S5 Table Frequency of attribute levels of gross income (per month). (DOCX)

S6 Table Frequency of attribute levels of overtime (per month). (DOCX)

S7 Table Frequency of attribute levels of promotion prospects. (DOCX)

S8 Table Frequency of attribute levels of diversity management. (DOCX)

S9 Table Frequency of attribute levels of LGBTQ*-friendly work climate. (DOCX)

S10 Table Correlation analysis of attributes across the 36 choice scenarios. Significance levels: * p<0.05, ** p<0.01, *** p<0.001.
(DOCX)

S11 Table Results from model comparison (complete model). Significance levels: * p<0.05, ** p<0.01, *** p<0.001;^1^ Reference value; Note: The estimated coefficients in column CL are from the conditional logit (CL) model that assumes homogenous preferences for individuals. The estimated coefficients in column MXL are from the mixed logit model (MXL) that accounts for the individual heterogeneity. In the MXL, all attributes except income, overtime, and promotion are random; Source: LGBielefeld 2021; own calculations.
(DOCX)

S12 Table Results from MXL sexual orientation. Significance levels: * p<0.05, ** p<0.01, *** p<0.001; ^1^ Reference value; Note: MXL stands for mixed logit model; Source: LGBielefeld 2021; own calculations.
(DOCX)

S13 Table Results from MXL gender identity. Significance levels: * p<0.05, ** p<0.01, *** p<0.001; ^1^ Reference value; Note: MXL stands for mixed logit model. Source: LGBielefeld 2021; own calculations.
(DOCX)

S14 Table Control–MXL discriminatory experiences. Significance levels: * p<0.05, ** p<0.01, *** p<0.001; ^1^ Reference value; Note: MXL stands for mixed logit model. Source: LGBielefeld 2021; own calculations.
(DOCX)

S15 Table Control–MXL age groups. Significance levels: * p<0.05, ** p<0.01, *** p<0.001; ^1^ Reference value; Note: MXL stands for mixed logit model. Source: LGBielefeld 2021; own calculations.
(DOCX)

S16 Table Control–MXL occupational status. Significance levels: * p<0.05, ** p<0.01, *** p<0.001; ^1^ Reference value; Note: MXL stands for mixed logit model. Source: LGBielefeld 2021; own calculations.
(DOCX)

S17 Table Control–MXL incl. interactions. Significance levels: * p<0.05, ** p<0.01, *** p<0.001; ^1^ Reference value; Note: MXL stands for mixed logit model. Source: LGBielefeld 2021; own calculations. DM = Diversity management, WC = Work climate.
(DOCX)

S18 Table Control–MXL without opt-out choices. Significance levels: * p<0.05, ** p<0.01, *** p<0.001; ^1^ Reference value; Note: MXL stands for mixed logit model. Source: LGBielefeld 2021; own calculations.
(DOCX)

S19 Table Control–MXL with only first three choices. Significance levels: * p<0.05, ** p<0.01, *** p<0.001; ^1^ Reference value; Note: MXL stands for mixed logit model. Source: LGBielefeld 2021; own calculations.
(DOCX)

## References

[1] OECD. Society at a Glance 2019. OECD Social Indicators. A Spotlight on LGBT People. Paris: Organisation for Economic Co-operation and Development; 2019.

[2] Valfort MA. OECD Social, Employment and Migration Working Papers. LGBTI in OECD Countries; 2017.

[3] Mendos LR, Botha K, Lelis RC, La Peña EL de, Savelev I Tan D. State-Sponsored Homophobia 2020: Global Legislation Overview Update; 2020.

[4] Flores AR, Langton L, Meyer IH, Romero AP. Victimization rates and traits of sexual and gender minorities in the United States: Results from the National Crime Victimization Survey, 2017. Sci Adv. 2020; 6. Epub 2020 Oct 2. doi: 10.1126/sciadv.aba6910 . 33008905 PMC7852385

[5] Flores AR. Social Acceptance of LGBTI People in 175 Countries And Locations. 1981 to 2020.UCLA; 2021. Available from: https://williamsinstitute.law.ucla.edu/wp-content/uploads/Global-Acceptance-Index-LGBTI-Nov-2021.pdf.

[6] FRA European Union Agency for Fundamental Rights. A long way to go for LGBTI equality; 2020. Available from: https://fra.europa.eu/sites/default/files/fra_uploads/fra-2020-lgbti-equality-1_en.pdf.

[7] de Vries L, Fischer M, Kasprowski D, Kroh M, Kühne S, Richter D, et al. LGBTQI* People on the Labor Market: Highly Educated, Frequently Discriminated Against. DIW—Deutsches Institut für Wirtschaftsforschung; Berlin 2020; DIW Weekly Report 36.

[8] Neumark D. Experimental Research on Labor Market Discrimination. Journal of Economic Literature. 2018;56:799–866. doi: 10.1257/jel.20161309

[9] Flage A. Discrimination against gays and lesbians in hiring decisions: a meta-analysis. IJM. 2020;41:671–91. doi: 10.1108/IJM-08-2018-0239

[10] Granberg M, Andersson PA, Ahmed A. Hiring Discrimination Against Transgender People: Evidence from a Field Experiment. Labour Economics. 2020;65:101860. doi: 10.1016/j.labeco.2020.101860

[11] Badgett ML, Carpenter CS, Sansone D. LGBTQ Economics. Journal of Economic Perspectives. 2021;35:141–70. doi: 10.1257/jep.35.2.141

[12] Drydakis N. Sexual orientation and earnings: a meta-analysis 2012–2020. J Popul Econ. 2021. doi: 10.1007/s00148-021-00862-1

[13] Kroh M, Kühne S, Kipp C, Richter D. Income, Social Support Networks, Life Satisfaction: Lesbians, Gays, and Bisexuals in Germany. Berlin; 2017. DIW Weekly Report 35.

[14] Finnigan R. Rainbow-Collar Jobs? Occupational Segregation by Sexual Orientation in the United States. Socius. 2020; 6:237802312095479. doi: 10.1177/2378023120954795

[15] Plug E, Webbink D, Martin N. Sexual Orientation, Prejudice, and Segregation. Journal of Labor Economics. 2014; 32:123–59. doi: 10.1086/673315

[16] Antecol H, Jong A, Steinberger M. The Sexual Orientation Wage Gap: The Role of Occupational Sorting and Human Capital. ILR Review. 2008;61:518–43. doi: 10.1177/001979390806100405

[17] Ellis L, Ratnasingam M, Wheeler M. Gender, sexual orientation, and occupational interests: Evidence of their interrelatedness. Personality and Individual Differences. 2012;53:64–9. doi: 10.1016/j.paid.2012.02.008

[18] Chung Y, Harmon LW. The Career Interests and Aspirations of Gay Men: How Sex-Role Orientation Is Related. Journal of Vocational Behavior. 1994;45:223–39. doi: 10.1006/jvbe.1994.1033

[19] Ng ES, Schweitzer L, Lyons ST. Anticipated Discrimination and a Career Choice in Nonprofit. Review of Public Personnel Administration. 2012;32:332–52. doi: 10.1177/0734371X12453055

[20] Lippa RA. Gender-related traits of heterosexual and homosexual men and women. Arch Sex Behav. 2002;31:83–98. doi: 10.1023/a:1014035302843 11910795

[21] Pager D, Pedulla DS. Race, self-selection, and the job search process. AJS. 2015;120:1005–54. doi: 10.1086/681072 . 26046224 PMC4651212

[22] Goldsmith AH, Sedo S, Darity W, Hamilton D. The labor supply consequences of perceptions of employer discrimination during search and on-the-job: Integrating neoclassical theory and cognitive dissonance. Journal of Economic Psychology. 2004; 25:15–39. doi: 10.1016/S0167-4870(02)00210-6

[23] Warner M. Introduction: Fear of a Queer Planet. Social Text. 1991; 29: 3–17.

[24] Hennesey R. Profit and Pleasure Sexual Identities in Late Capitalism. 2nd ed. New York: Routledge; 2017.

[25] Drydakis N. Sexual Orientation and Earnings. A Meta-Analysis 2012–2020. SSRN Journal. 2021. doi: 10.2139/ssrn.3874368

[26] Carpenter CS, Eppink ST, Gonzales G. Transgender Status, Gender Identity, and Socioeconomic Outcomes in the United States. ILR Review. 2020;73:573–99. doi: 10.1177/0019793920902776

[27] Ciprikis K, Cassells D, Berrill J. Transgender labour market outcomes: Evidence from the United States. Gender Work Organ. 2020;27:1378–401. doi: 10.1111/gwao.12501

[28] Schilt K, Wiswall M. Before and After: Gender Transitions, Human Capital, and Workplace Experiences. The B.E. Journal of Economic Analysis & Policy. 2008;8. doi: 10.2202/1935-1682.1862

[29] Frank J. Gay Glass Ceilings. Economica. 2006;73:485–508. doi: 10.1111/j.1468-0335.2006.00516.x

[30] Ahmed A, Andersson L, Hammarstedt M. Sexual orientation and occupational rank. Economics Bulletin. 2011;31:2422–33.

[31] Aksoy CG, Carpenter CS, Frank J, Huffman ML. Gay glass ceilings: Sexual orientation and workplace authority in the UK. Journal of Economic Behavior & Organization. 2019;159:167–80. doi: 10.1016/j.jebo.2019.01.013

[32] Bridges S, Mann S. Sexual Orientation, Legal Partnerships and Wages in Britain. Work, Employment and Society. 2019;33:1020–38. doi: 10.1177/0950017019873265

[33] de Vries L, Steinmetz S. Sexual Orientation, Workplace Authority and Occupational Segregation: Evidence from Germany. Work, Employment and Society. 2023. 10.1177/09500170231158513

[34] Bryant-Lees KB, Kite ME. Evaluations of LGBT job applicants: consequences of applying “out.” EDI. 2021;40:874–91. doi: 10.1108/EDI-01-2019-0048

[35] Frost DM, Meyer IH. Minority stress theory: Application, critique, and continued relevance. Current Opinion in Psychology. 2023; 51. doi: 10.1016/j.copsyc.2023.101579 37270877 PMC10712335

[36] Meyer IH. Prejudice, social stress, and mental health in lesbian, gay, and bisexual populations: conceptual issues and research evidence. Psychological Bulletin. 2003;129:674–97. doi: 10.1037/0033-2909.129.5.674 . 12956539 PMC2072932

[37] Pöge K, Dennert G, Koppe U, Güldenring A, Matthigack EB, Rommel A. Die gesundheitliche Lage von lesbischen, schwulen, bisexuellen sowie trans- und intergeschlechtlichen Menschen. Robert Koch-Institut; 2020. German.

[38] Kasprowski D, Fischer M, Chen X, de Vries L, Kroh M, Kühne S, et al. LGBTQI* People in Germany Face Staggering Health Disparities. DIW—Deutsches Institut für Wirtschaftsforschung; Berlin 2021; DIW Weekly Report 5 + 6.

[39] de Vries L. Diversität oder Diskriminierung im Management. Arbeit. 2021;30:215–37. doi: 10.1515/arbeit-2021-0016

[40] Drydakis N. Effect of Sexual Orientation on Job Satisfaction: Evidence from Greece. Ind Relat. 2015;54:162–87. doi: 10.1111/irel.12080

[41] Ragins BR. Sexual Orientation in the Workplace: The Unique Work and Career Experiences of Gay, Lesbian and Bisexual Workers. Bingley: Emerald (MCB UP); 2004; 35–120.

[42] Chung YB. Work Discrimination and Coping Strategies: Conceptual Frameworks for Counseling Lesbian, Gay, and Bisexual Clients. The Career Development Quarterly. 2001;50:33–44. doi: 10.1002/j.2161-0045.2001.tb00887.x

[43] Sansone D, Carpenter CS. Turing’s children: Representation of sexual minorities in STEM. PLoS One. 2020; 15:e0241596. Epub 2020 Nov 18. doi: 10.1371/journal.pone.0241596 . 33206668 PMC7673532

[44] Hughes BE. Coming out in STEM: Factors affecting retention of sexual minority STEM students. Sci Adv. 2018; 4: eaao6373. Epub 2018 Mar 14. doi: 10.1126/sciadv.aao6373 . 29546240 PMC5851677

[45] Konrad AM, Ritchie E, Lieb P, Corrigall E, Ritchie JE, et al. Sex Differences and Similarities in Job Attribute Preferences. A Meta-Analysis. Psychological Bulletin. 2000;126:593–641. doi: 10.1037/0033-2909.126.4.593 . 10900998

[46] Esser I, Lindh A. Job Preferences in Comparative Perspective 1989–2015: A Multidimensional Evaluation of Individual and Contextual Influences. International Journal of Sociology. 2018;48:142–69. doi: 10.1080/00207659.2018.1446118

[47] Griffith J, Combs GM. Racial Differences In Job Attribute Preferences: The Role Of Ethnic Identity And Self-Efficacy. AMPROC. 2015;2015:15401. doi: 10.5465/ambpp.2015.15401abstract

[48] Wiswall M, Zafar B. Preference for the Workplace, Investment in Human Capital, and Gender. Q J Econ. 2018; 33:457–507. Epub 2017 Aug 26. doi: 10.1093/qje/qjx035 . 30237622 PMC6141045

[49] Valet P, Sauer C, Tolsma J. Preferences for work arrangements: A discrete choice experiment. PLoS One. 2021; 16:e0254483. Epub 2021 Jul 12. doi: 10.1371/journal.pone.0254483 . 34252148 PMC8274907

[50] Frohn D, Meinhold F, Schmidt C. „Out im Office?!”Sexuelle Identität und Geschlechtsidentität, (Anti-)Diskriminierung und Diversity am Arbeitsplatz. Köln; 2017. German.

[51] Badgett ML, Durso LE, Mallory C, Kastanis A. The Business Impact of LGBT-Supportive Workplace Policies. 2013. Available from: https://escholarship.org/uc/item/3vt6t9zx.

[52] Holman EG, Fish JN, Oswald RF, Goldberg A. Reconsidering the LGBT Climate Inventory: Understanding Support and Hostility for LGBTQ Employees in the Workplace. J Career Assess. 2019; 27(3): 544–559. doi: 10.1177/1069072718788324 33967571 PMC8100868

[53] Wright T, Colgan F, Creegany C, McKearney A. Lesbian, gay and bisexual workers: equality, diversity and inclusion in the workplace. Equal Opportunities International. 2006;25:465–70. doi: 10.1108/02610150610713782

[54] Colgan F, Creegan C, McKearney A, Wright T. Lesbian, Gay and Bisexual Workers: Equality, Diversity and Inclusion in the Workplace. COERC, London Metropolitan University; 2006.

[55] Lloren A, Parini L. How LGBT-Supportive Workplace Policies Shape the Experience of Lesbian, Gay Men, and Bisexual Employees. Sex Res Soc Policy. 2017;14:289–99. doi: 10.1007/s13178-016-0253-x

[56] Allen SH, Mendez SN. Hegemonic Heteronormativity: Toward a New Era of Queer Family Theory. Journal of Family Theory & Review. 2018; 10(1): 70–86. 10.1111/jftr.12241

[57] Oswald RF, Blume LB, Marks SR. Decentering Heteronormativity: A Model for Family Studies. In: Bengtson VL, Acock AC, Allen KR, Dilworth-Anderson P, Klein DM editors. Sourcebook of Family Theory and Research. SAGE Publications, Inc; 2005. p 143–165.

[58] Aksoy CG, Carpenter CS, Frank J. Sexual Orientation and Earnings: New Evidence from the United Kingdom. ILR Review. 2018;71(1): 242–272. 10.1177/0019793916687759

[59] Jost M, Möser S. Salary, flexibility or career opportunity? A choice experiment on gender specific job preferences. Sec. Work, Employment and Organizations. 2023; 8. doi: 10.3389/fsoc.2023.1154324 37139224 PMC10150105

[60] Konrad AM, Corrigall E, Lieb P, Ritchie JE. Sex Differences in Job Attribute Preferences Among Managers and Business Students. Group & Organization Management. 2000; 25(2): 108–131. 10.1177/1059601100252002

[61] Zindel Z, de Vries L, Kühne S, Kroh M, Kasprowski D, Fischer M, et al. LGBielefeld 2021. Data report: Online survey on LGBTQI* people in Germany. 2023. 10.4119/unibi/2964217

[62] Fischer MM, Kroh M, de Vries L, Kasprowski D, Kühne S, Richter D, et al. Sexual and Gender Minority (SGM) Research Meets Household Panel Surveys: Research Potentials of the German Socio-Economic Panel and Its Boost Sample of SGM Households. European Sociological Review. 2022;38:321–35. doi: 10.1093/esr/jcab050

[63] Lancaster K. Consumer demand. A new approach. New York: Columbia University Press; 1971.

[64] Lancaster KJ. A New Approach to Consumer Theory. Journal of Political Economy. 1966;74:132–57.

[65] McFadden D. Conditional logit analysis of qualitative choice behavior. In: Zarembka P, editor. Frontiers in econometrics. New York: Academic Press; 1974. p. 105–42.

[66] Shrestha RM, Shrestha S, Sapkota VP. Evaluation of Job Preference of Prospective Dentists using Discrete Choice Experiment. Combined Issue. Econ J Dev Issues. 2015;19 & 20:100–19. doi: 10.3126/ejdi.v19i1-2.17707

[67] Kolstad JR. How to make rural jobs more attractive to health workers. Findings from a discrete choice experiment in Tanzania. Health Econ. 2011;20:196–211. doi: 10.1002/hec.1581 . 20094993

[68] Meng D, Xu G, He L, Zhang M, Padula WV, Davidson PM. Nursing students’ perceived value of the work environment. A discrete choice experiment. Geriatric Nursing. 2021;42:94–8. Epub 2020 Dec 16. doi: 10.1016/j.gerinurse.2020.12.002 . 33340916

[69] Janger J, Nowotny K. Job choice in academia. Research Policy. 2016;45:1672–83. doi: 10.1016/j.respol.2016.05.001

[70] Campbell D, Erdem S. Including Opt-Out Options in Discrete Choice Experiments: Issues to Consider. Patient. 2019;12:1–14. doi: 10.1007/s40271-018-0324-6 . 30073482

[71] Hole AR. DCREATE: Stata module to create efficient designs for discrete choice experiments. Statistical Software Components. 2015. Available from: https://ideas.repec.org/c/boc/bocode/s458059.html.

[72] McFadden D, Train K. Mixed MNL models for discrete response. J Appl Econ. 2000;15:447–70. doi: 10.1002/1099-1255(200009/10)15

[73] Revelt D, Train K. Mixed Logit with Repeated Choices: Households’ Choices of Appliance Efficiency Level. Review of Economics and Statistics. 1998;80:647–57. doi: 10.1162/003465398557735

[74] Hauber AB, Gonzales JM, Groothuis-Oudshoorn CGM, Prior T, Marshall DA, Cunningham C, et al.: Statistical Methods for the Analysis of Discrete Choice Experiments: A Report of the ISPOR Conjoint Analysis Good Research Practices Task Force. Value in health. 2016; 19(4), 300–315. doi: 10.1016/j.jval.2016.04.004 27325321

[75] Hole AR. Fitting Mixed Logit Models by Using Maximum Simulated Likelihood. The Stata Journal. 2007;7:388–401. doi: 10.1177/1536867X0700700306

[76] Lancsar E, Fiebig DG, Hole AR. Discrete Choice Experiments: A Guide to Model Specification, Estimation and Software. Pharmacoeconomics. 2017;35:697–716. doi: 10.1007/s40273-017-0506-4 . 28374325

[77] Train K. Discrete choice methods with simulation. 2nd ed. Cambridge, New York: Cambridge University Press; 2009.

[78] Malhotra NK. Marketing Research: An Applied Orientation. 7th ed. Pearson Education Limited; 2019.

