# Supplementary figures and images for: Career sacrifice for an LGBTQ*-friendly work environment? a choice experiment to investigate the job preferences of LGBTQ* people
Source: PLoS One. 2024 Jun 24;19(6):e0296419. doi: 10.1371/journal.pone.0296419 (PMC11195964; doi:10.1371/journal.pone.0296419)

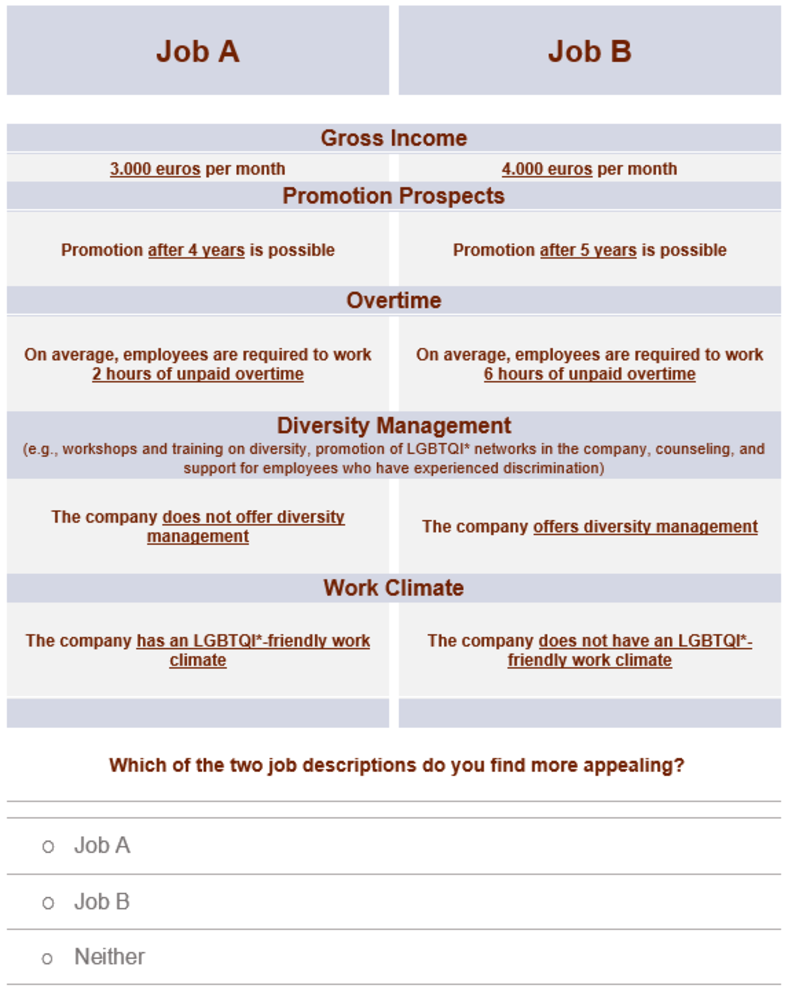

Supplement: S1 Fig — Notes: Translated to English. (TIF) [file pone.0296419.s001.tif]

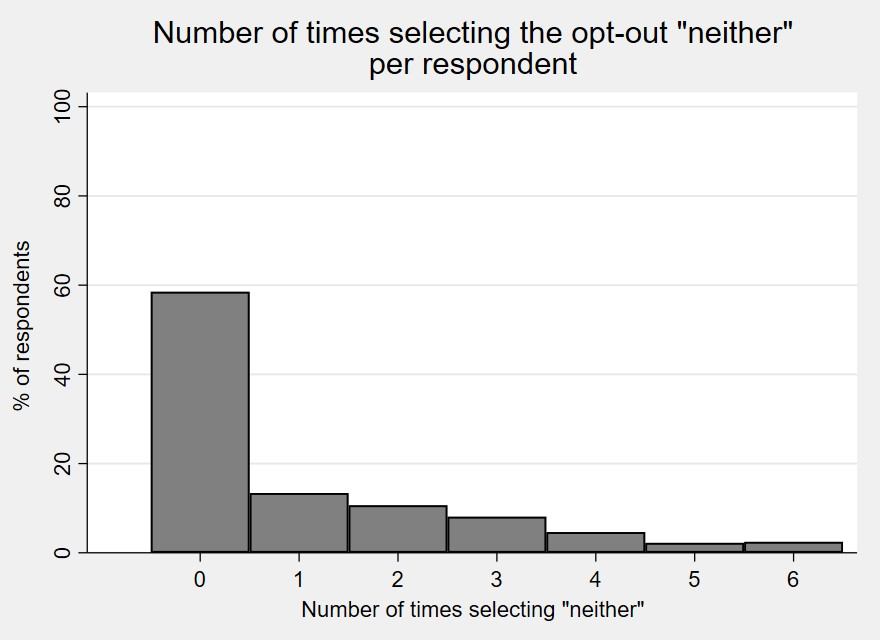

Supplement: S2 Fig — Notes: N = 4,505; Source: LGBielefeld 2021, own calculations. (TIF) [file pone.0296419.s002.tif]

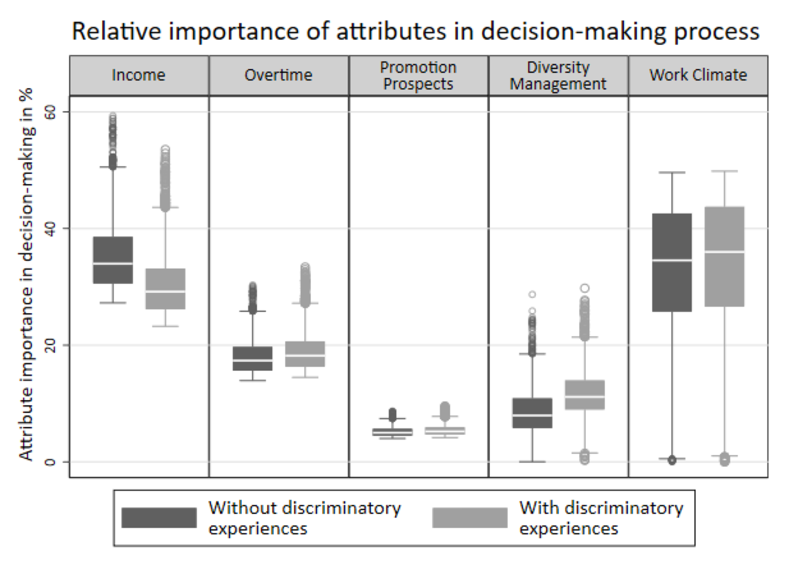

Supplement: S3 Fig — Notes: Without discriminatory experiences: N = 766; With discriminatory experiences: N = 3,735; Source: LGBielefeld 2021; own calculations. (TIF) [file pone.0296419.s003.tif]

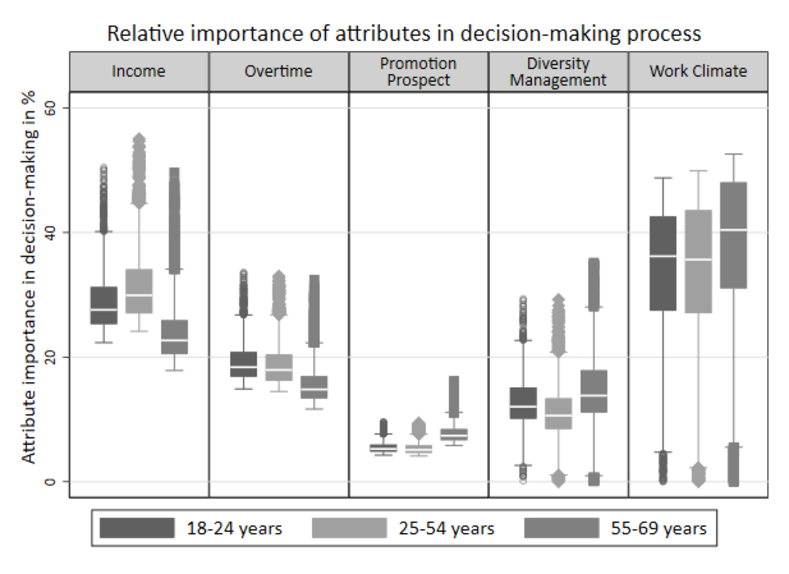

Supplement: S4 Fig — Notes: 18–24 years: N = 459; 24–54: N = 4,505; 55–69 years: N = 293; Source: LGBielefeld 2021; own calculations. (TIF) [file pone.0296419.s004.tif]

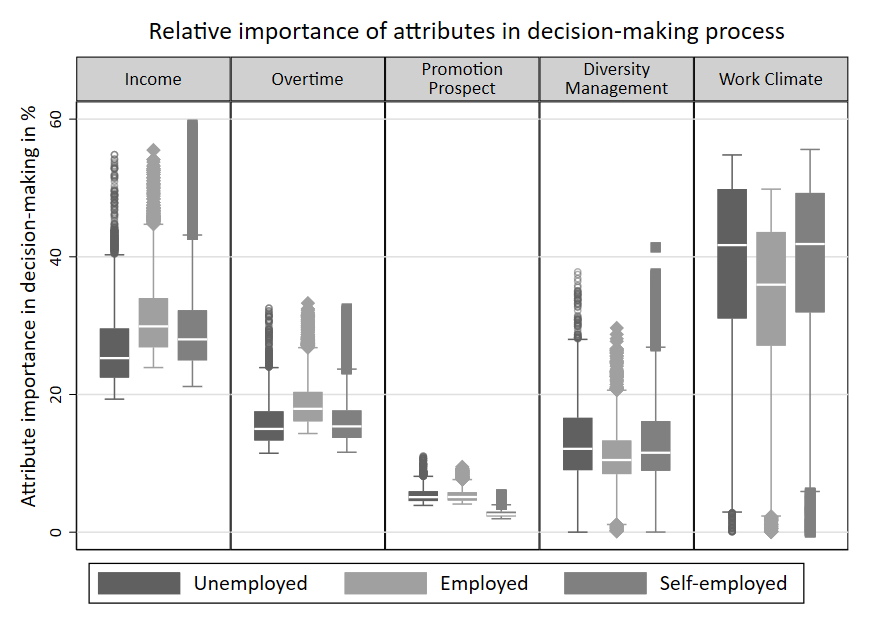

Supplement: S5 Fig — Notes: Unemployed: N = 626; Employed: N = 4,505; Self-employed: N = 465; Source: LGBielefeld 2021; own calculations. (TIF) [file pone.0296419.s005.tif]
