# Supplementary material for: Career sacrifice for an LGBTQ*-friendly work environment? a choice experiment to investigate the job preferences of LGBTQ* people
Source: PLoS One. 2024 Jun 24;19(6):e0296419. doi: 10.1371/journal.pone.0296419 (PMC11195964; doi:10.1371/journal.pone.0296419)
Supplement: S1 Table — Source: LGBielefeld 2021 (unweighted); SOEP v38.1 (weighted), own calculations. (DOCX) [file pone.0296419.s006.docx]

**S1 Table. Characteristics of participants in the** **LGBielefeld 2021 study and the Socio-Economic Panel (SOEP).**

|  | **LGBielefeld**  **2021** | | **SOEP**  **(LGBTQ*)** | | **SOEP**  **(cis-heterosexual)** | |
| --- | --- | --- | --- | --- | --- | --- |
|  | **Obs.** | **Mean** | **Obs.** | **Mean** | **Obs.** | **Mean** |
| Age | 4,507 | 35.80 | 374 | 37.85 | 6,616 | 40.10 |
| Children in household  (1 = yes) | 4,482 | 0.14 | 374 | 0.19 | 6,616 | 0.52 |
| Partner (1 = yes) | 4,465 | 0.75 | 345 | 0.57 | 5,847 | 0.76 |
| East Germany (1 = yes) | 4,503 | 0.11 | 374 | 0.26 | 6,615 | 0.20 |
| University degree (1 = yes) | 4,507 | 0.47 | 344 | 0.34 | 5,839 | 0.32 |
| Gross income | 3,495 | 3,579.63 | 276 | 3183.58 | 4,974 | 3422.42 |
| Net income | 3,964 | 2,337.36 | 276 | 2139.32 | 4,974 | 2237.59 |
| Contracted weekly working hours | 4,019 | 35.73 | 256 | 35.57 | 4,697 | 34.42 |
| Actual weekly working hours | 3,919 | 38.97 | 273 | 37.83 | 4,884 | 37.31 |
| General life satisfaction | 4,360 | 7.10 | 344 | 7.04 | 5,835 | 7.44 |

Source: LGBielefeld 2021 (unweighted); SOEP v38.1 (weighted), own calculations.
