# Supplementary material for: Career sacrifice for an LGBTQ*-friendly work environment? a choice experiment to investigate the job preferences of LGBTQ* people
Source: PLoS One. 2024 Jun 24;19(6):e0296419. doi: 10.1371/journal.pone.0296419 (PMC11195964; doi:10.1371/journal.pone.0296419)
Supplement: S2 Table — Source: LGBielefeld 2021. (DOCX) [file pone.0296419.s007.docx]

**S2 Table. Measurement of sexual orientation and gender/sex.**

|  | **Sexual orientation** | **Sex assigned at birth** | **Gender identity** |
| --- | --- | --- | --- |
| **Question** | How would you describe yourself: Are you…? | What sex were you assigned at birth, on your birth certificate | And which gender do you identify with today? |
| **Answer categories** | 1.) homosexual (gay or lesbian, i.e., attracted to your own gender)  2.) bisexual (attracted to your own gender and at least one other gender)  3.) pansexual (attracted to people regardless of their gender)  4.) heterosexual (attracted to another gender)  5.) asexual (not sexually attracted to other people)  6.) other sexual orientation not listed here [+ open-response format] | 1.) male  2.) female | 1.) male  2.) female  3.) non-binary  4.) trans*  5.) other gender, which is not listed here [+ open-response format]  6.) no gender |

Source: LGBielefeld 2021.
