# Supplementary material for: Career sacrifice for an LGBTQ*-friendly work environment? a choice experiment to investigate the job preferences of LGBTQ* people
Source: PLoS One. 2024 Jun 24;19(6):e0296419. doi: 10.1371/journal.pone.0296419 (PMC11195964; doi:10.1371/journal.pone.0296419)
Supplement: S3 Table — Notes: Overall N = 4,507; N = 19 missing information for sexual orientation not included in table; Source: LGBielefeld 2021, own calculations. (DOCX) [file pone.0296419.s008.docx]

**S3 Table. Frequencies of sexual orientation in analysis sample.**

| Sexual orientation | **Freq.** | **%** | **Cum. %** |
| --- | --- | --- | --- |
| *Lesbian or gay* | 3,274 | 72.95 | 72.95 |
| *Bi- or pansexual* | 1,028 | 22.91 | 95.86 |
| *Other orientation* | 186 | 4.14 | 100.00 |
| Total | 4,488 | 100.00 |  |

Notes: Overall N = 4,507; N =19 missing information for sexual orientation not included in table; Source: LGBielefeld 2021, own calculations.
