# Supplementary material for: Career sacrifice for an LGBTQ*-friendly work environment? a choice experiment to investigate the job preferences of LGBTQ* people
Source: PLoS One. 2024 Jun 24;19(6):e0296419. doi: 10.1371/journal.pone.0296419 (PMC11195964; doi:10.1371/journal.pone.0296419)
Supplement: S4 Table — Notes: Overall N = 4,507; N = 153 missing information for gender identity not included in table; Source: LGBielefeld 2021, own calculations. (DOCX) [file pone.0296419.s009.docx]

**S4 Table.** **Frequencies of gender identity in analysis sample.**

| Gender identity | **Freq.** | **%** | **Cum. %** |
| --- | --- | --- | --- |
| *Cisgender men of the LGBTQ* community* | 1,718 | 39.46 | 39.46 |
| *Cisgender women of the LGBTQ* community* | 2,172 | 49.89 | 89.34 |
| *Transgender, non-binary and other* | 464 | 10.66 | 100.00 |
| Total | 4,354 | 100.00 |  |

Notes: Overall N = 4,507; N =153 missing information for gender identity not included in table; Source: LGBielefeld 2021, own calculations.
