# Supplementary material for: Career sacrifice for an LGBTQ*-friendly work environment? a choice experiment to investigate the job preferences of LGBTQ* people
Source: PLoS One. 2024 Jun 24;19(6):e0296419. doi: 10.1371/journal.pone.0296419 (PMC11195964; doi:10.1371/journal.pone.0296419)
Supplement: S5 Table — (DOCX) [file pone.0296419.s010.docx]

**S5 Table. Frequency of attribute levels of gross income (per month).**

| Gross income (per month) | **Freq.** | **%** | **Cum. %** |
| --- | --- | --- | --- |
| *3,000 €* | 10 | 13.89 | 13.89 |
| *3,500 €* | 16 | 22.22 | 36.11 |
| *4,000 €* | 16 | 22.22 | 58.33 |
| *4,500 €* | 15 | 20.83 | 79.17 |
| *5,000 €* | 15 | 20.83 | 100.00 |
| Total | 72 | 100.00 |  |
