# Supplementary material for: Career sacrifice for an LGBTQ*-friendly work environment? a choice experiment to investigate the job preferences of LGBTQ* people
Source: PLoS One. 2024 Jun 24;19(6):e0296419. doi: 10.1371/journal.pone.0296419 (PMC11195964; doi:10.1371/journal.pone.0296419)
Supplement: S7 Table — (DOCX) [file pone.0296419.s012.docx]

**S7 Table. Frequency of attribute levels of promotion prospects.**

| Promotion prospects | **Freq.** | **%** | **Cum. %** |
| --- | --- | --- | --- |
| *After 3 years* | 19 | 26.39 | 26.39 |
| *After 4 years* | 27 | 37.50 | 63.89 |
| *After 5 years* | 26 | 36.11 | 100.00 |
| Total | 72 | 100.00 |  |
|  | | | |
