# Supplementary material for: Career sacrifice for an LGBTQ*-friendly work environment? a choice experiment to investigate the job preferences of LGBTQ* people
Source: PLoS One. 2024 Jun 24;19(6):e0296419. doi: 10.1371/journal.pone.0296419 (PMC11195964; doi:10.1371/journal.pone.0296419)
Supplement: S8 Table — (DOCX) [file pone.0296419.s013.docx]

**S8 Table. Frequency of attribute levels of diversity management.**

| Diversity management | **Freq.** | **%** | **Cum. %** |
| --- | --- | --- | --- |
| *Yes* | 36 | 50.00 | 50.00 |
| *No* | 36 | 50.00 | 100.00 |
| Total | 72 | 100.00 |  |
|  | | | |
