# Supplementary material for: Career sacrifice for an LGBTQ*-friendly work environment? a choice experiment to investigate the job preferences of LGBTQ* people
Source: PLoS One. 2024 Jun 24;19(6):e0296419. doi: 10.1371/journal.pone.0296419 (PMC11195964; doi:10.1371/journal.pone.0296419)
Supplement: S10 Table — Significance levels: * p<0.05, ** p<0.01, *** p<0.001. (DOCX) [file pone.0296419.s015.docx]

**S10 Table. Correlation analysis of attributes across the 36 choice scenarios.**

| Variables | (1) | (2) | (3) | (4) | (5) |
| --- | --- | --- | --- | --- | --- |
| (1) Gross income (per month) | 1.000 |  |  |  |  |
| (2) Overtime (per month) | -0.179 | 1.000 |  |  |  |
| (3) Promotion prospects | -0.104 | -0.191 | 1.000 |  |  |
| (4) Diversity Management | -0.093 | -0.105 | -0.124 | 1.000 |  |
| (5) LGBTQ*-friendly work climate | -0.114 | -0.140 | -0.124 | -0.111 | 1.000 |

Significance levels: * p<0.05, ** p<0.01, *** p<0.001.
