# Supplementary material for: Career sacrifice for an LGBTQ*-friendly work environment? a choice experiment to investigate the job preferences of LGBTQ* people
Source: PLoS One. 2024 Jun 24;19(6):e0296419. doi: 10.1371/journal.pone.0296419 (PMC11195964; doi:10.1371/journal.pone.0296419)
Supplement: S11 Table — Significance levels: * p<0.05, ** p<0.01, *** p<0.001;1 Reference value; Note: The estimated coefficients in column CL are from the conditional logit (CL) model that assumes homogenous preferences for individuals. The estimated coefficients in column MXL are from the mixed logit model (MXL) that accounts for the individual heterogeneity. In the MXL, all attributes except income, overtime, and promotion are random; Source: LGBielefeld 2021; own calculations. (DOCX) [file pone.0296419.s016.docx]

**S11 Table.** **Results from model comparison (complete model).**

|  | **CL** | | **MXL** | |
| --- | --- | --- | --- | --- |
|  | **Coef.** | **SE** | **Coef.** | **SE** |
| **Main** | | | | |
| Income | | | | |
| 3,000 €^1^ -0.893 -1.551 | | | | |
| 3,500 € | -0.575^***^ | 0.021 | -0.870^***^ | 0.035 |
| 4,000 € | 0.221^***^ | 0.024 | 0.444^***^ | 0.035 |
| 4,500 € | 0.424^***^ | 0.023 | 0.688^***^ | 0.037 |
| 5,000 € | 0.823^***^ | 0.022 | 1.289^***^ | 0.038 |
| Overtime | | | | |
| 0 hours^1^ 0.487 0.700 | | | | |
| 2 hours | 0.165^***^ | 0.014 | 0.301^***^ | 0.022 |
| 6 hours | -0.652^***^ | 0.023 | -1.001^***^ | 0.038 |
| Promotion prospects | | | | |
| 3 years^1^ 0.061 -0.015 | | | | |
| 4 years | 0.131^***^ | 0.017 | 0.250^***^ | 0.027 |
| 5 years | -0.192^***^ | 0.018 | -0.235^***^ | 0.027 |
| Diversity management | 0.295^***^ | 0.011 | 0.499^***^ | 0.018 |
| Work climate | 1.017^***^ | 0.016 | 1.655^***^ | 0.036 |
| ASC*block1 | 0.163^***^ | 0.063 | 0.485^***^ | 0.367 |
| ASC*block2 | 0.268^***^ | 0.094 | 0.663^***^ | 0.269 |
| ASC*block3 | 0.243^***^ | 0.092 | 0.741^***^ | 0.209 |
| ASC*block4 | 0.499^***^ | 0.092 | 1.526^***^ | 0.263 |
| ASC*block5 | 0.111^***^ | 0.097 | 0.249^***^ | 0.164 |
| ASC | -0.275^***^ | 0.096 | -0.749^***^ | 0.150 |
| **SD** | | | | |
| Diversity Management |  |  | -0.380^***^ | 0.030 |
| Work Climate |  |  | 1.020^***^ | 0.027 |
| ASC*block1 |  |  | 1.156^***^ | 1.042 |
| ASC*block2 |  |  | 1.348^***^ | 0.638 |
| ASC*block3 |  |  | 1.575^***^ | 0.388 |
| ASC*block4 |  |  | 2.598^***^ | 0.286 |
| ASC*block5 |  |  | 0.477^***^ | 0.151 |
| ASC |  |  | 2.356^***^ | 0.193 |
| Log-likelihood (full model) | -19754.82 | | -16544.94 | |
| Prob. > chi2 | 0.0000 | | 0.0000 | |
| AIC | 39541.65 | | 33137.88 | |
| BIC | 39690.45 | | 33361.10 | |
| Respondents | 4505 | | 4505 | |
| Job descriptions | 80862 | | 80862 | |

Significance levels: * p<0.05, ** p<0.01, *** p<0.001;^1^ Reference value; Note: The estimated coefficients in column CL are from the conditional logit (CL) model that assumes homogenous preferences for individuals. The estimated coefficients in column MXL are from the mixed logit model (MXL) that accounts for the individual heterogeneity. In the MXL, all attributes except income, overtime, and promotion are random; Source: LGBielefeld 2021; own calculations.
