# Supplementary material for: Career sacrifice for an LGBTQ*-friendly work environment? a choice experiment to investigate the job preferences of LGBTQ* people
Source: PLoS One. 2024 Jun 24;19(6):e0296419. doi: 10.1371/journal.pone.0296419 (PMC11195964; doi:10.1371/journal.pone.0296419)
Supplement: S12 Table — Significance levels: * p<0.05, ** p<0.01, *** p<0.001; 1 Reference value; Note: MXL stands for mixed logit model; Source: LGBielefeld 2021; own calculations. (DOCX) [file pone.0296419.s017.docx]

**S12 Table.** **Results from MXL sexual orientation****.**

|  | **Full model** | | **Lesbian or gay** | | **Bi- or pansexual** | |
| --- | --- | --- | --- | --- | --- | --- |
|  | **Coef.** | **SE** | **Coef.** | **SE** | **Coef.** | **SE** |
| **Main** | | | | | | |
| Income | | | | | | |
| 3,000 €^1^ -1.551 -1.630 -1.358 | | | | | | |
| 3,500 € | -0.870^***^ | 0.035 | -0.892^***^ | 0.041 | -0.815^***^ | 0.071 |
| 4,000 € | 0.444^***^ | 0.035 | 0.463^***^ | 0.041 | 0.378^***^ | 0.075 |
| 4,500 € | 0.688^***^ | 0.037 | 0.744^***^ | 0.044 | 0.59^***^ | 0.074 |
| 5,000 € | 1.289^***^ | 0.038 | 1.315^***^ | 0.044 | 1.205^***^ | 0.078 |
| Overtime | | | | | | |
| 0 hours^1^ 0.700 0.643 0.770 | | | | | | |
| 2 hours | 0.301^***^ | 0.022 | 0.314^***^ | 0.026 | 0.266^***^ | 0.045 |
| 6 hours | -1.001^***^ | 0.038 | -0.958^***^ | 0.044 | -1.036^***^ | 0.077 |
| Promotion prospects | | | | | | |
| 3 years^1^ -0.015 -0.017 -0.009 | | | | | | |
| 4 years | 0.250^***^ | 0.027 | 0.270^***^ | 0.032 | 0.187^***^ | 0.055 |
| 5 years | -0.235^***^ | 0.027 | -0.253^***^ | 0.031 | -0.178^***^ | 0.057 |
| Diversity management | 0.499^***^ | 0.018 | 0.502^***^ | 0.020 | 0.488^***^ | 0.039 |
| Work climate | 1.655^***^ | 0.036 | 1.649^***^ | 0.042 | 1.622^***^ | 0.072 |
| ASC*block1 | 0.485^***^ | 0.367 | 0.229^***^ | 0.190 | 1.112^***^ | 0.651 |
| ASC*block2 | 0.663^***^ | 0.269 | 0.549^***^ | 0.244 | 0.938^***^ | 0.571 |
| ASC*block3 | 0.741^***^ | 0.209 | 0.832^***^ | 0.229 | 0.358^***^ | 0.387 |
| ASC*block4 | 1.526^***^ | 0.263 | 1.721^***^ | 0.305 | 1.078^***^ | 0.502 |
| ASC*block5 | 0.249^***^ | 0.164 | 0.42^***^ | 0.202 | -0.114^***^ | 0.376 |
| ASC | -0.749^***^ | 0.150 | -0.802^***^ | 0.143 | -0.756^***^ | 0.289 |
| **SD** | | | | | | |
| Diversity Management | -0.380^***^ | 0.030 | -0.334^***^ | 0.037 | -0.422^***^ | 0.063 |
| Work Climate | 1.020^***^ | 0.027 | 1.024^***^ | 0.031 | 0.990^***^ | 0.057 |
| ASC*block1 | 1.156^***^ | 1.042 | 0.340^***^ | 0.144 | 1.831^***^ | 0.401 |
| ASC*block2 | 1.348^***^ | 0.638 | 1.174^***^ | 0.457 | 2.099^***^ | 1.177 |
| ASC*block3 | 1.575^***^ | 0.388 | 1.541^***^ | 0.308 | 1.668^***^ | 0.843 |
| ASC*block4 | 2.598^***^ | 0.286 | -2.832^***^ | 0.293 | -2.432^***^ | 0.548 |
| ASC*block5 | 0.477^***^ | 0.151 | -0.297^***^ | 1.142 | -0.984^***^ | 0.443 |
| ASC | 2.356^***^ | 0.193 | 2.476^***^ | 0.092 | 1.953^***^ | 1.003 |
| Log-likelihood (full model) | -16544.94 | | -11961.354 | | -3803.6686 | |
| Prob. > chi2 | 0.0000 | | 0.0000 | | 0.0000 | |
| Respondents | 4505 | | 3273 | | 1027 | |
| Job descriptions | 80862 | | 58746 | | 18441 | |

Significance levels: * p<0.05, ** p<0.01, *** p<0.001; ^1^ Reference value; Note: MXL stands for mixed logit model; Source: LGBielefeld 2021; own calculations.
