# Supplementary material for: Career sacrifice for an LGBTQ*-friendly work environment? a choice experiment to investigate the job preferences of LGBTQ* people
Source: PLoS One. 2024 Jun 24;19(6):e0296419. doi: 10.1371/journal.pone.0296419 (PMC11195964; doi:10.1371/journal.pone.0296419)
Supplement: S13 Table — Significance levels: * p<0.05, ** p<0.01, *** p<0.001; 1 Reference value; Note: MXL stands for mixed logit model. Source: LGBielefeld 2021; own calculations. (DOCX) [file pone.0296419.s018.docx]

**S13 Table.** **Results from MXL gender identity.**

|  | **Full model** | | **Cisgender men of the LGBTQ* community** | | **Cisgender women of the LGBTQ* community** | | **Transgender, non-binary,  and other** | |
| --- | --- | --- | --- | --- | --- | --- | --- | --- |
|  | **Coef.** | **SE** | **Coef.** | **SE** | **Coef.** | **SE** | **Coef.** | **SE** |
| **Main** | | | | | | | | |
| Income | | | | | | | | |
| 3,000 €^1^ | -1.551^***^ |  | -1.587^***^ |  | -1.526^***^ |  | -1.428^***^ |  |
| 3,500 € | -0.870^***^ | 0.035 | -1.018^***^ | 0.059 | -0.824^***^ | 0.050 | -0.621^***^ | 0.101 |
| 4,000 € | 0.444^***^ | 0.035 | 0.389^***^ | 0.058 | 0.459^***^ | 0.051 | 0.561^***^ | 0.110 |
| 4,500 € | 0.688^***^ | 0.037 | 0.740^***^ | 0.059 | 0.719^***^ | 0.054 | 0.407^***^ | 0.113 |
| 5,000 € | 1.289^***^ | 0.038 | 1.476^***^ | 0.061 | 1.172^***^ | 0.055 | 1.081^***^ | 0.122 |
| Overtime | | | | | | | | |
| 0 hours^1^ | 0.700^***^ |  | 0.660^***^ |  | 0.727^***^ |  | 0.807^***^ |  |
| 2 hours | 0.301^***^ | 0.022 | 0.267^***^ | 0.037 | 0.320^***^ | 0.032 | 0.335^***^ | 0.074 |
| 6 hours | -1.001^***^ | 0.038 | -0.927^***^ | 0.058 | -1.047^***^ | 0.058 | -1.142^***^ | 0.122 |
| Promotion prospects | | |  |  |  |  |  |  |
| 3 years^1^ | -0.015^***^ |  | 0.114^***^ |  | -0.064^***^ |  | -0.145^***^ |  |
| 4 years | 0.250^***^ | 0.027 | 0.230^***^ | 0.043 | 0.253^***^ | 0.040 | 0.326^***^ | 0.090 |
| 5 years | -0.235^***^ | 0.027 | -0.344^***^ | 0.045 | -0.189^***^ | 0.038 | -0.181^***^ | 0.085 |
| Diversity management | 0.499^***^ | 0.018 | 0.436^***^ | 0.027 | 0.551^***^ | 0.027 | 0.522^***^ | 0.063 |
| Work climate | 1.655^***^ | 0.036 | 1.481^***^ | 0.053 | 1.767^***^ | 0.055 | 1.828^***^ | 0.127 |
| ASC*block1 | 0.485^***^ | 0.367 | 0.288^***^ | 0.268 | 0.633^***^ | 0.255 | 1.175^***^ | 0.705 |
| ASC*block2 | 0.663^***^ | 0.269 | 0.576^***^ | 0.299 | 0.834^***^ | 0.274 | 0.377^***^ | 0.666 |
| ASC*block3 | 0.741^***^ | 0.209 | 1.087^***^ | 0.376 | 0.684^***^ | 0.231 | 0.505^***^ | 0.463 |
| ASC*block4 | 1.526^***^ | 0.263 | 2.210^***^ | 0.706 | 1.278^***^ | 0.326 | 0.477^***^ | 0.558 |
| ASC*block5 | 0.249^***^ | 0.164 | 0.584^***^ | 0.335 | 0.428^***^ | 0.221 | -1.133^***^ | 0.455 |
| ASC | -0.749^***^ | 0.150 | -1.032^***^ | 0.214 | -0.515^**^ | 0.166 | -0.889^***^ | 0.353 |
| **SD** | | | | | | | | |
| Diversity Management | -0.380^***^ | 0.030 | 0.358^***^ | 0.047 | -0.372^***^ | 0.046 | -0.440^***^ | 0.125 |
| Work Climate | 1.020^***^ | 0.027 | 1.010^***^ | 0.041 | 1.011^***^ | 0.039 | 1.100^***^ | 0.100 |
| ASC*block1 | 1.156^***^ | 1.042 | -0.432^***^ | 0.449 | -1.000^***^ | 0.446 | 2.611^***^ | 0.765 |
| ASC*block2 | 1.348^***^ | 0.638 | 0.822^***^ | 0.383 | 1.526^***^ | 0.285 | 1.738^***^ | 0.724 |
| ASC*block3 | 1.575^***^ | 0.388 | 2.400^***^ | 0.441 | 0.715^***^ | 0.359 | -0.633^***^ | 0.306 |
| ASC*block4 | 2.598^***^ | 0.286 | 2.609^***^ | 1.230 | 2.817^***^ | 0.324 | 1.498^***^ | 0.586 |
| ASC*block5 | 0.477^***^ | 0.151 | 1.170^***^ | 0.550 | -0.136^***^ | 0.399 | -0.140^***^ | 0.172 |
| ASC | 2.356^***^ | 0.193 | 2.560^***^ | 0.183 | 2.303^***^ | 0.105 | 2.130^***^ | 0.203 |
| Log-likelihood  (full model) | -16544.94 | | -6292.8802 | | -7861.1517 | | -1695.2306 | |
| Prob. > chi2 | 0.0000 | | 0.0000 | | 0.0000 | | 0.0000 | |
| Respondents | 4505 | | 1717 | | 2171 | | 464 | |
| Job descriptions | 80862 | | 30828 | | 38964 | | 8,316 | |

Significance levels: * p<0.05, ** p<0.01, *** p<0.001; ^1^ Reference value; Note: MXL stands for mixed logit model. Source: LGBielefeld 2021; own calculations.
