# Supplementary material for: Career sacrifice for an LGBTQ*-friendly work environment? a choice experiment to investigate the job preferences of LGBTQ* people
Source: PLoS One. 2024 Jun 24;19(6):e0296419. doi: 10.1371/journal.pone.0296419 (PMC11195964; doi:10.1371/journal.pone.0296419)
Supplement: S14 Table — Significance levels: * p<0.05, ** p<0.01, *** p<0.001; 1 Reference value; Note: MXL stands for mixed logit model. Source: LGBielefeld 2021; own calculations. (DOCX) [file pone.0296419.s019.docx]

**S14 Table. Control – MXL discriminatory experiences.**

|  | **Full model** | | | **W/ discrim. exp.** | | | **W/o discrim. exp.** | | |
| --- | --- | --- | --- | --- | --- | --- | --- | --- | --- |
|  | **Coef.** |  | **SE** | **Coef.** |  | **SE** | **Coef.** |  | **SE** |
| **Main** | | | | | | | | | |
| Income | | | | | | | | | |
| 3,000 €^1^ | -1.551 |  |  | -1.522 |  |  | -1.685 |  |  |
| 3,500 € | -0.870 | ^***^ | 0.035 | -0.833 | ^***^ | 0.038 | -1.040 | ^***^ | 0.091 |
| 4,000 € | 0.444 | ^***^ | 0.035 | 0.430 | ^***^ | 0.039 | 0.478 | ^***^ | 0.082 |
| 4,500 € | 0.688 | ^***^ | 0.037 | 0.671 | ^***^ | 0.040 | 0.780 | ^***^ | 0.090 |
| 5,000 € | 1.289 | ^***^ | 0.038 | 1.254 | ^***^ | 0.041 | 1.467 | ^***^ | 0.094 |
| Overtime | | | | | | | | | |
| 0 hours^1^ | 0.690 |  |  | 0.700 |  |  | 0.706 |  |  |
| 2 hours | 0.301 | ^***^ | 0.022 | 0.318 | ^***^ | 0.025 | 0.230 | ^***^ | 0.053 |
| 6 hours | -1.001 | ^***^ | 0.038 | -1.024 | ^***^ | 0.042 | -0.920 | ^***^ | 0.086 |
| Promotion prospects | | | | | | | | | |
| 3 years^1^ | -0.015 |  |  | 0.010 |  |  | -0.136 |  |  |
| 4 years | 0.250 | ^***^ | 0.027 | 0.243 | ^***^ | 0.030 | 0.299 | ^***^ | 0.065 |
| 5 years | -0.235 | ^***^ | 0.027 | -0.253 | ^***^ | 0.030 | -0.163 | ^**^ | 0.061 |
| Diversity management | 0.499 | ^***^ | 0.018 | 0.534 | ^***^ | 0.020 | 0.351 | ^***^ | 0.041 |
| Work climate | 1.655 | ^***^ | 0.036 | 1.698 | ^***^ | 0.040 | 1.463 | ^***^ | 0.080 |
| ASC*block1 | 0.485 |  | 0.367 | 0.613 | ^**^ | 0.204 | 0.546 |  | 0.476 |
| ASC*block2 | 0.663 | ^*^ | 0.269 | 0.720 | ^**^ | 0.251 | 1.077 | ^*^ | 0.533 |
| ASC*block3 | 0.741 | ^***^ | 0.209 | 0.883 | ^***^ | 0.225 | 0.524 |  | 0.472 |
| ASC*block4 | 1.526 | ^***^ | 0.263 | 1.595 | ^***^ | 0.250 | 1.543 |  | 0.852 |
| ASC*block5 | 0.249 |  | 0.164 | 0.345 |  | 0.211 | 0.020 |  | 0.524 |
| ASC | -0.749 | ^***^ | 0.150 | -0.614 | ^***^ | 0.134 | -1.096 | ^***^ | 0.370 |
| **SD** | | | | | | | | | |
| Diversity Management | -0.380 | ^***^ | 0.030 | -0.368 |  | 0.033 | -0.385 | ^***^ | 0.078 |
| Work Climate | 1.020 | ^***^ | 0.027 | 1.028 |  | 0.030 | 0.996 | ^***^ | 0.064 |
| ASC*block1 | 1.156 |  | 1.042 | 1.571 |  | 0.131 | 0.075 |  | 0.172 |
| ASC*block2 | 1.348 | ^*^ | 0.638 | 1.630 | ^***^ | 0.364 | -1.899 | ^***^ | 0.305 |
| ASC*block3 | 1.575 | ^***^ | 0.388 | 1.852 | ^***^ | 0.391 | -0.308 | ^**^ | 0.107 |
| ASC*block4 | 2.598 | ^***^ | 0.286 | 2.750 | ^***^ | 0.241 | 2.439 | ^*^ | 1.055 |
| ASC*block5 | 0.477 | ^**^ | 0.151 | -0.926 |  | 0.219 | -0.247 |  | 0.830 |
| ASC | 2.356 | ^***^ | 0.193 | 2.075 | ^***^ | 0.476 | 3.101 | ^***^ | 0.231 |
| Log-likelihood (full model) | -16544.94 | | | -13615.455 | | | -2858.9468 | | |
| Prob. > chi2 | 0.0000 | | | 0.0000 | | | 0.0000 | | |
| Respondents | 4505 | | | 3735 | | | 766 | | |
| Job descriptions | 80862 | | | 67044 | | | 13746 | | |

Significance levels: * p<0.05, ** p<0.01, *** p<0.001; ^1^ Reference value; Note: MXL stands for mixed logit model. Source: LGBielefeld 2021; own calculations.
