# Supplementary material for: Career sacrifice for an LGBTQ*-friendly work environment? a choice experiment to investigate the job preferences of LGBTQ* people
Source: PLoS One. 2024 Jun 24;19(6):e0296419. doi: 10.1371/journal.pone.0296419 (PMC11195964; doi:10.1371/journal.pone.0296419)
Supplement: S15 Table — Significance levels: * p<0.05, ** p<0.01, *** p<0.001; 1 Reference value; Note: MXL stands for mixed logit model. Source: LGBielefeld 2021; own calculations. (DOCX) [file pone.0296419.s020.docx]

**S15 Table. Control – MXL age groups.**

|  | **18-24 years** | | | **25-54 years** | | | **55-69 years** | | |
| --- | --- | --- | --- | --- | --- | --- | --- | --- | --- |
|  | **Coef.** | | **SE** | **Coef.** | | **SE** | **Coef.** | | **SE** |
| **Main** |  |  |  |  |  |  |  |  |  |
| 3,000 €^1^ | -1.417 |  |  | -1.551^***^ |  |  | -0.989 |  |  |
| 3,500 € | -0.671 | ^***^ | 0.101 | -0.870 | ^***^ | 0.035 | -0.804 | ^***^ | 0.132 |
| 4,000 € | 0.422 | ^***^ | 0.111 | 0.444 | ^***^ | 0.035 | 0.292 | ^*^ | 0.121 |
| 4,500 € | 0.553 | ^***^ | 0.100 | 0.688 | ^***^ | 0.037 | 0.701 | ^***^ | 0.128 |
| 5,000 € | 1.113 | ^***^ | 0.106 | 1.289 | ^***^ | 0.038 | 0.800 | ^***^ | 0.135 |
| Overtime |  |  |  |  |  |  |  |  |  |
| 0 hours^1^ | 0.600 |  |  | 0.700 |  |  | 0.513 |  |  |
| 2 hours | 0.486 | ^***^ | 0.069 | 0.301 | ^***^ | 0.022 | 0.143 |  | 0.087 |
| 6 hours | -1.086 | ^***^ | 0.111 | -1.001 | ^***^ | 0.038 | -0.656 | ^***^ | 0.126 |
| Promotion prospects |  |  |  |  |  |  |  |  |  |
| 3 years^1^ | 0.090 |  |  | -0.015 |  |  | 0.012 |  |  |
| 4 years | 0.196 | ^*^ | 0.081 | 0.250 | ^***^ | 0.027 | 0.285 | ^**^ | 0.096 |
| 5 years | -0.286 | ^***^ | 0.080 | -0.235 | ^***^ | 0.027 | -0.297 | ^**^ | 0.113 |
| Diversity management | 0.561 | ^***^ | 0.058 | 0.499 | ^***^ | 0.018 | 0.567 | ^***^ | 0.073 |
| Work climate | 1.633 | ^***^ | 0.102 | 1.655 | ^***^ | 0.036 | 1.534 | ^***^ | 0.124 |
| ASC*block1 | 0.613 |  | 0.485 | 0.485 |  | 0.367 | 0.633 |  | 0.811 |
| ASC*block2 | -0.012 |  | 0.417 | 0.663 | ^*^ | 0.269 | 0.074 |  | 0.788 |
| ASC*block3 | 1.239 |  | 0.677 | 0.741 | ^***^ | 0.209 | 1.161 |  | 0.851 |
| ASC*block4 | 2.085 | ^*^ | 0.881 | 1.526 | ^***^ | 0.263 | 1.074 |  | 0.775 |
| ASC*block5 | -0.289 |  | 0.452 | 0.249 |  | 0.164 | 0.560 |  | 0.861 |
| ASC | -1.219 | ^***^ | 0.314 | -0.749 | ^***^ | 0.150 | -0.929 |  | 0.598 |
| **SD** |  |  |  |  |  |  |  |  |  |
| Diversity Management | 0.352 | ^***^ | 0.081 | -0.380 | ^***^ | 0.030 | -0.402 | ^***^ | 0.102 |
| Work Climate | 0.903 | ^***^ | 0.072 | 1.020 | ^***^ | 0.027 | 0.900 | ^***^ | 0.099 |
| ASC*block1 | 1.049 | ^*^ | 0.514 | 1.156 |  | 1.042 | -0.994 |  | 0.612 |
| ASC*block2 | 0.321 |  | 0.642 | 1.348 | ^*^ | 0.638 | -1.133 |  | 0.680 |
| ASC*block3 | 1.904 | ^***^ | 0.491 | 1.575 | ^***^ | 0.388 | 1.843 | ^***^ | 0.361 |
| ASC*block4 | 2.823 | ^**^ | 0.885 | 2.598 | ^***^ | 0.286 | -1.369 | ^**^ | 0.474 |
| ASC*block5 | 1.032 |  | 0.752 | 0.477 | ^**^ | 0.151 | -1.126 | ^*^ | 0.480 |
| ASC | 1.684 | ^***^ | 0.212 | 2.356 | ^***^ | 0.193 | 2.794 | ^***^ | 0.301 |
| Log-likelihood  (full model) | -1571.27 | | | -16544.94 | | | -1101.43 | | |
| Prob. > chi2 | 0.0000 | | | 0.0000 | | | 0.0000 | | |
| Respondents | 459 | | | 4505 | | | 293 | | |
| Job descriptions | 8259 | | | 80862 | | | 5214 | | |

Significance levels: * p<0.05, ** p<0.01, *** p<0.001; ^1^ Reference value; Note: MXL stands for mixed logit model. Source: LGBielefeld 2021; own calculations.
