# Supplementary material for: Career sacrifice for an LGBTQ*-friendly work environment? a choice experiment to investigate the job preferences of LGBTQ* people
Source: PLoS One. 2024 Jun 24;19(6):e0296419. doi: 10.1371/journal.pone.0296419 (PMC11195964; doi:10.1371/journal.pone.0296419)
Supplement: S16 Table — Significance levels: * p<0.05, ** p<0.01, *** p<0.001; 1 Reference value; Note: MXL stands for mixed logit model. Source: LGBielefeld 2021; own calculations. (DOCX) [file pone.0296419.s021.docx]

**S16 Table. Control – MXL occupational status.**

|  | **Unemployed** | | | **Employed** | | | **Self-employed** | | |
| --- | --- | --- | --- | --- | --- | --- | --- | --- | --- |
|  | **Coef.** |  | **SE** | **Coef.** |  | **SE** | **Coef.** |  | **SE** |
| **Main** | | | | | | | | | |
| Income | | | | | | | | | |
| 3,000 €^1^ | -1.280 |  |  | -1.550 |  |  | -1.260 |  |  |
| 3,500 € | -0.692 | ^***^ | 0.094 | -0.870 | ^***^ | 0.035 | -0.665 | ^***^ | 0.101 |
| 4,000 € | 0.416 | ^***^ | 0.094 | 0.444 | ^***^ | 0.035 | 0.317 | ^**^ | 0.102 |
| 4,500 € | 0.526 | ^***^ | 0.091 | 0.688 | ^***^ | 0.037 | 0.605 | ^***^ | 0.105 |
| 5,000 € | 1.030 | ^***^ | 0.101 | 1.289 | ^***^ | 0.038 | 1.003 | ^***^ | 0.117 |
| Overtime | | | | | | | | | |
| 0 hours^1^ | 0.530 |  |  | 0.700 |  |  | 0.491 |  |  |
| 2 hours | 0.310 | ^***^ | 0.067 | 0.301 | ^***^ | 0.022 | 0.260 | ^***^ | 0.070 |
| 6 hours | -0.840 | ^***^ | 0.100 | -1.001 | ^***^ | 0.038 | -0.751 | ^***^ | 0.109 |
| Promotion prospects | | | | | | | | | |
| 3 years^1^ | -0.218 |  |  | -0.015 |  |  | -0.017 |  |  |
| 4 years | 0.247 | ^**^ | 0.077 | 0.250 | ^***^ | 0.027 | 0.113 |  | 0.088 |
| 5 years | -0.029 |  | 0.073 | -0.235 | ^***^ | 0.027 | -0.096 |  | 0.082 |
| Diversity management | 0.559 | ^***^ | 0.058 | 0.499 | ^***^ | 0.018 | 0.473 | ^***^ | 0.059 |
| Work climate | 1.927 | ^***^ | 0.118 | 1.655 | ^***^ | 0.036 | 1.641 | ^***^ | 0.114 |
| ASC*block1 | 0.856 |  | 0.596 | 0.485 |  | 0.367 | 0.233 |  | 0.567 |
| ASC*block2 | 0.899 |  | 0.582 | 0.663 | ^*^ | 0.269 | 0.237 |  | 0.618 |
| ASC*block3 | 1.039 |  | 0.582 | 0.741 | ^***^ | 0.209 | 0.943 |  | 0.588 |
| ASC*block4 | 1.591 | ^**^ | 0.599 | 1.526 | ^***^ | 0.263 | 1.376 | ^*^ | 0.656 |
| ASC*block5 | -0.387 |  | 0.440 | 0.249 | ^**^ | 0.164 | -0.064 |  | 0.574 |
| ASC | -0.579 |  | 0.331 | -0.749 | ^***^ | 0.150 | -0.709 |  | 0.422 |
| **SD** | | | | | | | | | |
| Diversity Management | -0.529 | ^***^ | 0.084 | -0.380 | ^***^ | 0.030 | 0.470 | ^***^ | 0.095 |
| Work Climate | 1.110 | ^***^ | 0.083 | 1.020 | ^***^ | 0.027 | 0.962 | ^***^ | 0.080 |
| ASC*block1 | -2.509 | ^***^ | 0.711 | 1.156 |  | 1.042 | -0.336 |  | 1.147 |
| ASC*block2 | 2.677 | ^**^ | 0.773 | 1.348 | ^*^ | 0.638 | -1.046 |  | 0.702 |
| ASC*block3 | 2.130 | ^***^ | 0.603 | 1.575 | ^***^ | 0.388 | 1.452 | ^***^ | 0.372 |
| ASC*block4 | 1.699 | ^*^ | 0.792 | 2.598 | ^***^ | 0.286 | 3.684 | ^***^ | 0.608 |
| ASC*block5 | -1.112 | ^*^ | 0.538 | 0.477 | ^**^ | 0.151 | 0.735 |  | 0.893 |
| ASC | 2.349 | ^***^ | 0.256 | 2.356 | ^***^ | 0.193 | 2.855 | ^***^ | 0.228 |
| Log-likelihood (full model) | -2267.85 | | | -16544.94 | | | -1781.79 | | |
| Prob. > chi2 | 0.0000 | | | 0.0000 | | | 0.0000 | | |
| Respondents | 626 | | | 4505 | | | 465 | | |
| Job descriptions | 11250 | | | 80862 | | | 8343 | | |

Significance levels: * p<0.05, ** p<0.01, *** p<0.001; ^1^ Reference value; Note: MXL stands for mixed logit model. Source: LGBielefeld 2021; own calculations.
