# Supplementary material for: Career sacrifice for an LGBTQ*-friendly work environment? a choice experiment to investigate the job preferences of LGBTQ* people
Source: PLoS One. 2024 Jun 24;19(6):e0296419. doi: 10.1371/journal.pone.0296419 (PMC11195964; doi:10.1371/journal.pone.0296419)
Supplement: S17 Table — Significance levels: * p<0.05, ** p<0.01, *** p<0.001; 1 Reference value; Note: MXL stands for mixed logit model. Source: LGBielefeld 2021; own calculations. DM = Diversity management, WC = Work climate. (DOCX) [file pone.0296419.s022.docx]

**S17 Table. Control – MXL incl. interactions.**

|  | | **Full model** | | | **w/ all interactions** | | |
| --- | --- | --- | --- | --- | --- | --- | --- |
|  | | **Coef,** | **SE** | | **Coef,** | **SE** | |
| **Main** | |  |  | |  |  | |
| Income | |  |  |  |  |  |  |
| 3,000 €^1^ | | -1.551 |  |  | -1.584 |  |  |
| 3.500 € | | -0.870 | ^***^ | 0.035 | -0.894 | ^***^ | 0.039 |
| 4.000 € | | 0.444 | ^***^ | 0.035 | 0.443 | ^***^ | 0.039 |
| 4.500 € | | 0.688 | ^***^ | 0.037 | 0.712 | ^***^ | 0.042 |
| 5.000 € | | 1.289 | ^***^ | 0.038 | 1.324 | ^***^ | 0.043 |
| Overtime | |  |  |  |  |  |  |
| 0 hours^1^ | | 0.700 |  |  | 0.722 |  |  |
| 2 hours | | 0.301 | ^***^ | 0.022 | 0.319 | ^***^ | 0.025 |
| 6 hours | | -1.001 | ^***^ | 0.038 | -1.041 | ^***^ | 0.043 |
| Promotion prospects | |  |  |  |  |  |  |
| 3 years^1^ | | -0.015 |  |  | -0.042 |  |  |
| 4 years | | 0.250 | ^***^ | 0.027 | 0.265 | ^***^ | 0.030 |
| 5 years | | -0.235 | ^***^ | 0.027 | -0.223 | ^***^ | 0.030 |
| Diversity management | | 0.499 | ^***^ | 0.018 | 0.558 | ^***^ | 0.114 |
| Work climate | | 1.655 | ^***^ | 0.036 | 2.125 | ^***^ | 0.174 |
| Age x | DM |  |  |  | 0.000 |  | 0.002 |
|  | WC |  |  |  | 0.006 |  | 0.003 |
| Child x | DM |  |  |  | 0.058 |  | 0.048 |
|  | WC |  |  |  | 0.097 |  | 0.075 |
| Partnership x | DM |  |  |  | 0.033 |  | 0.038 |
|  | WC |  |  |  | 0.006 |  | 0.055 |
| East Germany x | DM |  |  |  | 0.013 |  | 0.052 |
|  | WC |  |  |  | 0.029 |  | 0.078 |
| Education level x | DM |  |  |  | -0.005 |  | 0.034 |
|  | WC |  |  |  | 0.010 |  | 0.051 |
| Net income x | DM |  |  |  | 0.000 | ^*^ | 0.000 |
|  | WC |  |  |  | 0.000 | ^***^ | 0.000 |
| Contract. weekly working hours x | DM |  |  |  | 0.000 |  | 0.002 |
|  | WC |  |  |  | -0.007 |  | 0.004 |
| ASC*block1 |  | 0.485 |  | 0.367 | 0.670 |  | 0.415 |
| ASC*block2 |  | 0.663 | ^*^ | 0.269 | 0.688 | ^**^ | 0.237 |
| ASC*block3 |  | 0.741 | ^***^ | 0.209 | 0.910 | ^***^ | 0.254 |
| ASC*block4 |  | 1.526 | ^***^ | 0.263 | 1.791 | ^***^ | 0.283 |
| ASC*block5 |  | 0.249 |  | 0.164 | 0.385 | ^*^ | 0.189 |
| ASC |  | -0.749 | ^***^ | 0.150 | -0.620 | ^***^ | 0.158 |
| **SD** |  |  |  |  |  |  |  |
| Diversity Management | | -0.380 | ^***^ | 0.030 | 0.325 | ^***^ | 0.038 |
| Work Climate |  | 1.020 | ^***^ | 0.027 | 1.006 | ^***^ | 0.031 |
| ASC*block1 |  | 1.156 |  | 1.042 | 1.141 |  | 1.300 |
| ASC*block2 |  | 1.348 | ^*^ | 0.638 | 1.308 | ^***^ | 0.375 |
| ASC*block3 |  | 1.575 | ^***^ | 0.388 | 1.563 | ^***^ | 0.400 |
| ASC*block4 |  | 2.598 | ^***^ | 0.286 | -2.752 | ^***^ | 0.264 |
| ASC*block5 |  | 0.477 | ^**^ | 0.151 | 0.517 |  | 0.374 |
| ASC |  | 2.356 | ^***^ | 0.193 | 2.264 | ^***^ | 0.194 |
| Log-likelihood (fullmodel) | | -16,544.94 | | | -12,914.13 | | |
| Prob, > chi2 | | 0.00 | | | 0.00 | | |
| AIC |  | 33,137.88 | | | 25,904.27 | | |
| BIC |  | 33,361.10 | | | 26,248.72 | | |
| Respondents | | 4,505 | | | 3,554 | | |
| Job descriptions | | 80,862 | | | 63,873 | | |

Significance levels: * p<0.05, ** p<0.01, *** p<0.001; ^1^ Reference value; Note: MXL stands for mixed logit model. Source: LGBielefeld 2021; own calculations. DM = Diversity management, WC = Work climate.
