# Supplementary material for: Career sacrifice for an LGBTQ*-friendly work environment? a choice experiment to investigate the job preferences of LGBTQ* people
Source: PLoS One. 2024 Jun 24;19(6):e0296419. doi: 10.1371/journal.pone.0296419 (PMC11195964; doi:10.1371/journal.pone.0296419)
Supplement: S19 Table — Significance levels: * p<0.05, ** p<0.01, *** p<0.001; 1 Reference value; Note: MXL stands for mixed logit model. Source: LGBielefeld 2021; own calculations. (DOCX) [file pone.0296419.s024.docx]

**S19 Table. Control – MXL with only first three choices.**

|  |  | **Full model** | | **First 3 choices** | | |
| --- | --- | --- | --- | --- | --- | --- |
|  | **Coef.** |  | **SE** | **Coef.** |  | **SE** |
| **Main** | | | | | | |
| Income | | | | | | |
| 3,000 €^1^ | -1.551 |  |  | -1.744 |  |  |
| 3,500 € | -0.870 | ^***^ | 0.035 | -0.546 | ^***^ | 0.061 |
| 4,000 € | 0.444 | ^***^ | 0.035 | 0.391 | ^***^ | 0.049 |
| 4,500 € | 0.688 | ^***^ | 0.037 | 0.714 | ^***^ | 0.067 |
| 5,000 € | 1.289 | ^***^ | 0.038 | 1.184 | ^***^ | 0.065 |
| Overtime | | | | | | |
| 0 hours^1^ | 0.700 |  |  | 0.667 |  |  |
| 2 hours | 0.301 | ^***^ | 0.022 | -0.014 |  | 0.046 |
| 6 hours | -1.001 | ^***^ | 0.038 | -0.654 | ^***^ | 0.057 |
| Promotion prospects | | | | | | |
| 3 years^1^ | -0.015 |  |  | 0.254 |  |  |
| 4 years | 0.250 | ^***^ | 0.027 | 0.123 | ^*^ | 0.048 |
| 5 years | -0.235 | ^***^ | 0.027 | -0.377 | ^***^ | 0.047 |
| Diversity management | 0.499 | ^***^ | 0.018 | 0.614 | ^***^ | 0.029 |
| Work climate | 1.655 | ^***^ | 0.036 | 1.656 | ^***^ | 0.056 |
| ASC*block1 | 0.485 |  | 0.367 | 0.594 | ^*^ | 0.277 |
| ASC*block2 | 0.663 | ^*^ | 0.269 | 0.151 |  | 0.204 |
| ASC*block3 | 0.741 | ^***^ | 0.209 | 0.271 |  | 0.179 |
| ASC*block4 | 1.526 | ^***^ | 0.263 | 1.550 | ^***^ | 0.287 |
| ASC*block5 | 0.249 |  | 0.164 | 0.367 |  | 0.272 |
| ASC | -0.749 | ^***^ | 0.150 | -1.093 | ^***^ | 0.141 |
| **SD** | | | | | | |
| Diversity Management | -0.380 | ^***^ | 0.030 | 0.425 | ^***^ | 0.063 |
| Work Climate | 1.020 | ^***^ | 0.027 | 0.885 | ^***^ | 0.047 |
| ASC*block1 | 1.156 |  | 1.042 | 2.121 | ^***^ | 0.375 |
| ASC*block2 | 1.348 | ^*^ | 0.638 | 0.465 |  | 0.495 |
| ASC*block3 | 1.575 | ^***^ | 0.388 | 0.314 |  | 0.506 |
| ASC*block4 | 2.598 | ^***^ | 0.286 | 3.010 | ^***^ | 0.308 |
| ASC*block5 | 0.477 | ^**^ | 0.151 | 1.665 | ^***^ | 0.435 |
| ASC | 2.356 | ^***^ | 0.193 | 2.134 | ^***^ | 0.129 |
| Log-likelihood (full model) | -16544.94 | | | -8429.37 | | |
| Prob. > chi2 | 0.0000 | | | 0 | | |
| Respondents | 4505 | | | 4505 | | |
| Job descriptions | 80862 | | | 40506 | | |

Significance levels: * p<0.05, ** p<0.01, *** p<0.001; ^1^ Reference value; Note: MXL stands for mixed logit model. Source: LGBielefeld 2021; own calculations.
